# Supplementary material for: The endogenous Coxiella burnetii plasmid encodes a functional toxin–antitoxin system
Source: Mol Microbiol. 2022 Nov 28;118(6):744–64. doi: 10.1111/mmi.15001 (PMC10098735; doi:10.1111/mmi.15001)
Supplement: Supplementary file 1 — Supinfo S1 [file MMI-118-744-s001.pdf]

Title: The endogenous *Coxiella burnetii* plasmid encodes a functional toxin-antitoxin system.

Authors: Shaun Wachter[ab], Diane C Cockrell[c], Heather E Miller[d], Kimmo Virtaneva[e], Kishore Kanakabandi[e], Benjamin Darwitz[f], Robert A Heinzen[a] and Paul A Beare[ae]*

* Corresponding author: Paul Beare, pbeare@niaid.nih.gov,

Supplementary Information

Figure S1_IcmD CRISPRi knockdown and complement

Figure S2_Double knockdown of IcmD and ScvA

Figure S3_ACCM CRISPRi

Figure S4_THP1 CRISPRi

Figure S5_c-myc blots

Figure S6_Effector knockdowns

Figure S7_CBUA0027 knockdown

Figure S8_cbuA0028-cbuA0028G alignment

Figure S9_Pulldown-lysate samples

Figure S10_EMSA of PCBUA0028-PCBUA0029

Figure S11_cbuA0027 promoters in cbuA0028G

Figure S12_Off targeting sgRNAs

Figure S13_TA systems in C. burnetii

Table S1 - Stains and plasmids using in this study

Table S2 - Primers used in this study

Supplementary file 1

Supplementary Figure legends

Figure S1 – Inducible knockdown of IcmD production by CRISPR interference and complementation using codon optimized *icmD*. (a) Sequences of wild-type (*icmD*) and codon optimized (*icmD-CO*) icmD. Sequences targeting *icmD* target A and target B are denoted in blue and red, respectively. Silent mutations made to the *icmD-CO* nucleotide sequence are denoted in bold nucleotides. PAM sequences are underlined. (b) Schematic of the aTc-inducible expression of *icmD*-target A-CO. The orientation of the *tetR* repressor, P*tetA* promoters and *icmD*-target A-CO codon optimized gene are depicted. (c) Immunoblots of *icmD* CRISPRi strains containing the pJB-TetRA-icmD-CO complementation plasmid in the absence of CRISPRi inducer (IPTG) or complement inducer (aTc). Specific protein detection was achieved using anti-IcmK, anti-cmyc (dCas9) and anti-IcmD antibodies. Expression of codon optimized Target A *icmD* restored production of IcmD in the *icmD* CRISPRi Target A strain but was unable to restore IcmD production in the *icmD* CRISPRi Target B strain.

Figure S2 – Dual CRISPRi repression of IcmD and ScvA translation. (a) Schematic of the IPTG-inducible double CRISPRi expression construct (from pB-CRISPRi-*icmD*-sgRNA-1-*scvA*-sgRNA-1). (b) Immunoblots of the *icmD*/*scvA* double CRISPRi knockdown strain in the absence or presence of IPTG. Specific protein detection was achieved using anti-c-Myc (dCas9), anti-IcmD, anti-ScvA, and anti-IcmK, antibodies. Induction of CRISPRi resulted in dual knockdown of both IcmD and ScvA production.

Figure S3 – CRISPRi knockdown of QpH1 ORFs in axenic media. Individual *C. burnetii* CRISPRi strains targeting one of two separate regions of each ORF of QpH1 were grown ACCM-D in the absence (CRISPRi off) or presence of 0.1 mM IPTG (CRISPRi on) for 6 days. Replication is shown as fold increase in *C. burnetii* genome equivalents (GE) of CRISPRi strains. Results are

expressed as the means of results from two biological replicates from three independent experiments. Error bars indicate the standard deviations from the means, and asterisks indicate a statistically significant difference (Student's *t* test, * - $P < 0.05$, ** - $P < 0.01$ *** - $P < 0.001$, and **** - $P < 0.0001$) compared to values for the CRISPRi off samples.

Figure S4 – CRISPRi knockdown of QpH1 ORFs in THP-1 macrophages. Individual *C. burnetii* CRISPRi strains targeting one of three separate regions of each ORF of QpH1 were grown in the absence (CRISPRi off) or presence of 2 mM IPTG (CRISPRi on) for 5 days. Replication is shown as fold increase in *C. burnetii* genome equivalents (GE) of CRISPRi strains. Results are expressed as the means of results from two biological replicates from three independent experiments. Error bars indicate the standard deviations from the means, and asterisks indicate a statistically significant difference (Student's *t* test, * - $P < 0.05$, ** - $P < 0.01$ *** - $P < 0.001$, and **** - $P < 0.0001$) compared to values for the CRISPRi off samples.

Figure S5 – Detection of dCas9 induction in *C. burnetii* QpH1 CRISPRi strains. Lysates of *C. burnetii* QpH1 CRISPRi strains grown for 7 days in ACCM-D with or without 0.1 mM IPTG were analyzed by immunoblot. Production of dCas9 was detected using an anti-c-myc antibody. The dCas9 protein was detected in all induced strains except for the *antitoxP* CRISPRi strains. CRISPRi knockdown of *antitoxP* causes severe growth defects in axenic media, hence no dCas9 protein was detected.

Figure S6 – QPCR analysis of CRISPRi knockdown of QpH1 Dot/Icm substrate genes. RNA was extracted from QpH1 Dot/Icm substrate strains grown in ACCM-D for 6 days in the absence (CRISPRi off) or presence of IPTG (CRISPRi on). Samples for QPCR analysis were normalized using the comparative CT method. For each RNA sample a gene specific or control *groEL* probe and primer set was used to examine the level of expression.

Figure S7 – QPCR analysis confirmed CRISPRi knockdown *antitoxP*. RNA was extracted from *antitoxP*-84 CRISPRi strain grown in ACCM-D for 6 days in the absence (CRISPRi off) or presence of IPTG (CRISPRi on). Samples for QPCR analysis were normalized using the comparative CT method. For each RNA sample a *antitoxP* specific or control *groEL* probe and rimer set was used to examine the level of expression.

Figure S8 – Alignment of *toxP* and *antitoxP* from NMII and G Q212. The *toxP* (a) and *antitoxP* (b) genes from NMII and G Q212 strains were aligned using clustal omega. The G Q212 genes are labeled *toxP*G and *antitoxP*G. The putative promoter (-35 and -10 regions) for *toxP* are denoted and the sequence underlined. The start codon for *toxP* and *toxP*G are colored red and blue, respectively. Asterisks demark conserved nucleotides.

Figure S9 – Production of AntitoxP, ToxP and CBU0665 by cell-free IVTT. Cell-free IVTT was carried out using the following combinations of template DNA (250 ng each): pDEST15-*antitoxP* (GST-A27), pET28a(+)-*toxP* (A28-V5), pEXP1-*cbu0665* (XpressT-665), GST-A27/A28-V5 or GST-A27/XpressT-665. Lysates from cell-free IVTT were analyzed by immunoblots probed (a) simultaneously with anti-V5 and anti-XpressT to detect ToxP and CBU0665, respectively, or with (b) anti-GST to detect AntitoxP.

Figure S10 – EMSA shows the AntitoxP/ToxP complex does not bind the *cbuA0029* or *groEL* promoters. EMSAs show interactions between biotin-labeled *toxP* (P*toxP*) or *cbuA0029* (P*cbuA0029*) promoters and increasing concentrations of purified AntitoxP/ToxP complex. Biotin-labeled P*groEL* with ToxP/AntitoxP (640 nM) was included as negative control. The location of bound and unbound probe is depicted by arrows.

Figure S11 - Alignment of the *toxP*-*antitoxP* operon from NMII and G Q212. The *toxP*-*antitoxP* operon from NMII and G Q212 strains were aligned using clustal omega. The G Q212 operon is labeled *toxP*G/*antitoxP*G. The putative sigma 70 promoters (-35 and -10 regions) are denoted, and the sequence underlined. The start codon for *toxP* and *toxPG* are colored red and blue, respectively. Bold italicized sequence denotes the *antitoxP* and *antitoxPG* start codons. The *antitoxP* P2 and P3 promoter sequences are colored green and grey, respectively. Asterisks demark conserved nucleotides. Only the *antitoxP* P3 promoter is conserved in both strains.

Figure S12 – Off-targeting of *cbuA0021*-sgRNAs. (a) Alignments of the CRISPRi targeted region of *cbuA0021* with *cbu0334* (*thiDE*) and *cbu0055* (*ubiA*). The location of the three CRISPRi target regions in demarked with arrows. Sequences colored red denote conserved regions within the 20 bp CRISPRi targets. (b) CRISRPi repression by *cbuA0021*-sgRNA-61 (CBUA0021-61) and *cbuA0021*-sgRNA-63 (CBUA0021-63) causes a growth defect in axenic media, whereas only *cbuA0021*-sgRNA-62 (CBUA0021-62) causes a growth defect in THP-1 macrophages. Replication is shown as fold increase in *C. burnetii* genome equivalents (GE) of CRISPRi strains targeting *cbuA0021* after 6 days in ACCM-D or after a 5-day infection of THP-1 macrophages in the absence (CRISPRi off) or presence (CRISPRi on) of CRISPRi system inducer molecule IPTG. Replication of a no sgRNA target was examined as a negative control. CRISPRi repression of the essential *icmD* Dot/Icm apparatus gene was used as a positive control in THP-1 macrophages. Results are expressed as the means of results from two biological replicates from three independent experiments. Error bars indicate the standard deviations from the means, and asterisks indicate a statistically significant difference (Student's t test, ** - $P < 0.01$, and *** - $P < 0.001$) compared to values for the CRISPRi off samples. (c) QPCR analysis confirmed CRISPRi knockdown *cbuA0021* and shows off-targeting knockdown on *cbuA0334*. RNA was extracted from *cbuA0021*-

61 CRISPRi strain grown in ACCM-D for 6 days in the absence (CRISPRi off) or presence of IPTG (CRISPRi on). Samples for QPCR analysis were normalized using the comparative CT method. For each RNA sample a *cbuA0021*- and *cbu0334*- specific or control *groEL* probe and primer set was used to examine the level of expression.

Figure S13 – Eleven TA systems were identified in *C. burnetii*. Schematic of the 11 TA modules in *C. burnetii* indicating their gene orientation. Black arrows indicate putative promoter regions in non-canonical TA modules for driving increased expression of the antitoxin module. Red arrows depict the location of 5′ truncations in other *C. burnetii* strains. Dark blue arrows represent *himar1* insertions that result in strong replication defects in Vero cells. The light blue arrow depicts a *himar1* mutant with a mild replication defect. The green arrow represents the location of a *himar1* mutant that has normal replication. Key: antitoxin and toxin genes are colored light and dark grey, respectively.

Supplemental figure 1

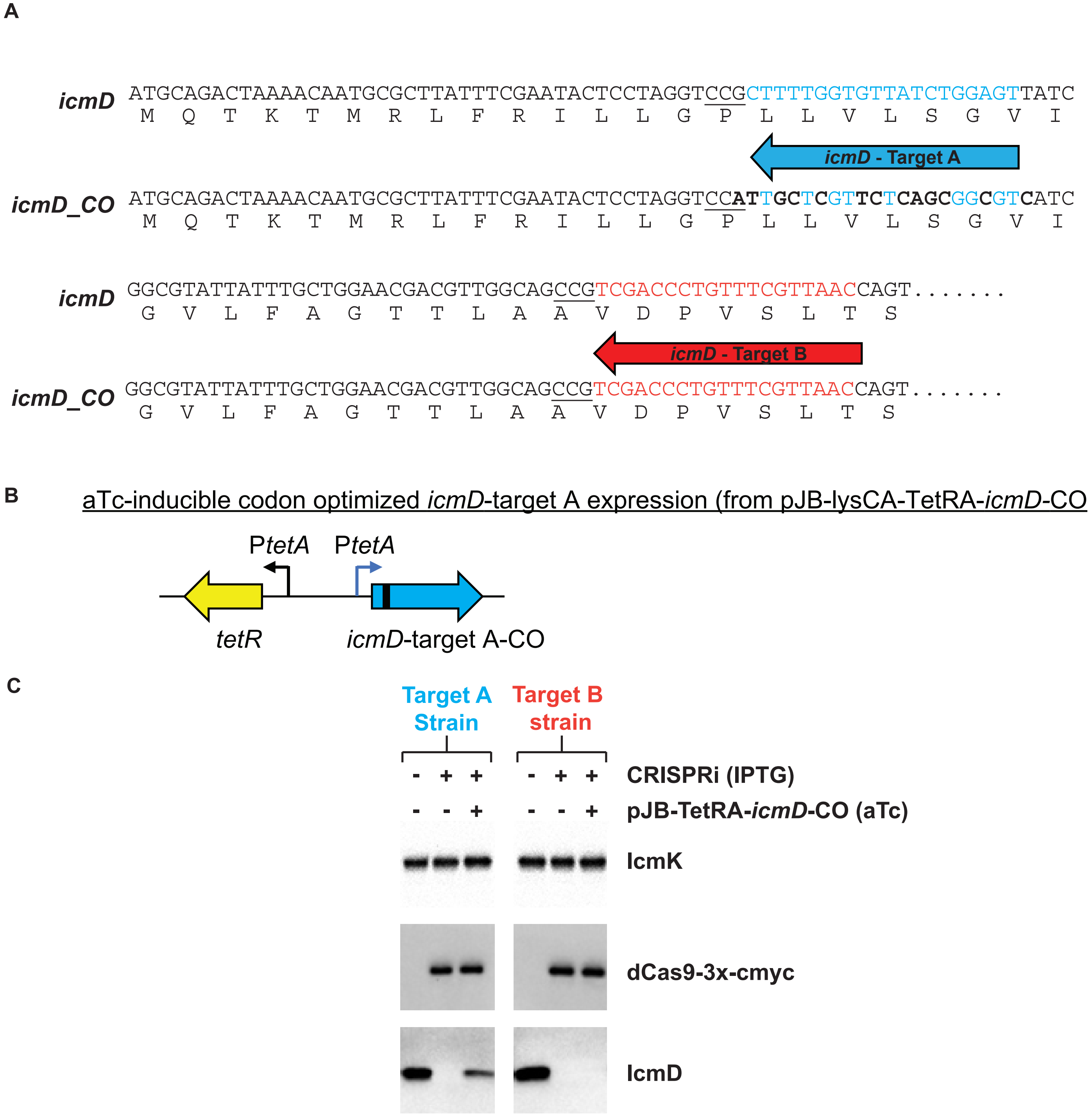

Supplemental figure 2

A

IPTG-inducible double CRISPRi expression (from pB-CRISPRi--icmD-sgRNA-1-ScvA-sgRNA-1)

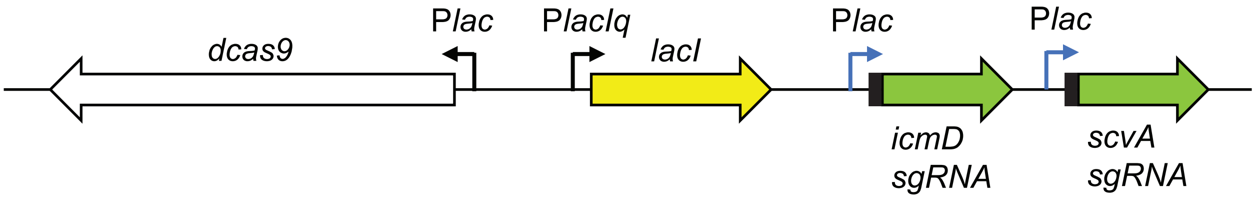

B

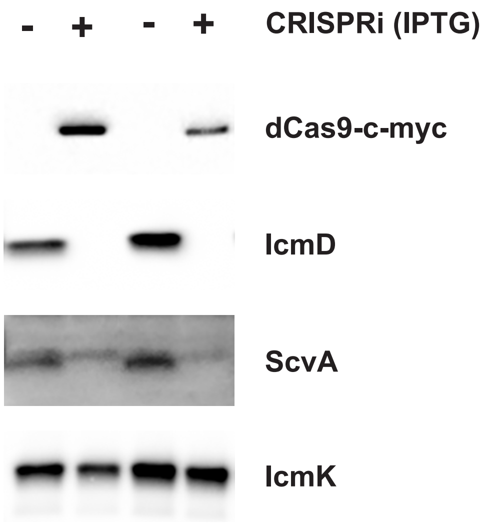

Supplemental figure 3

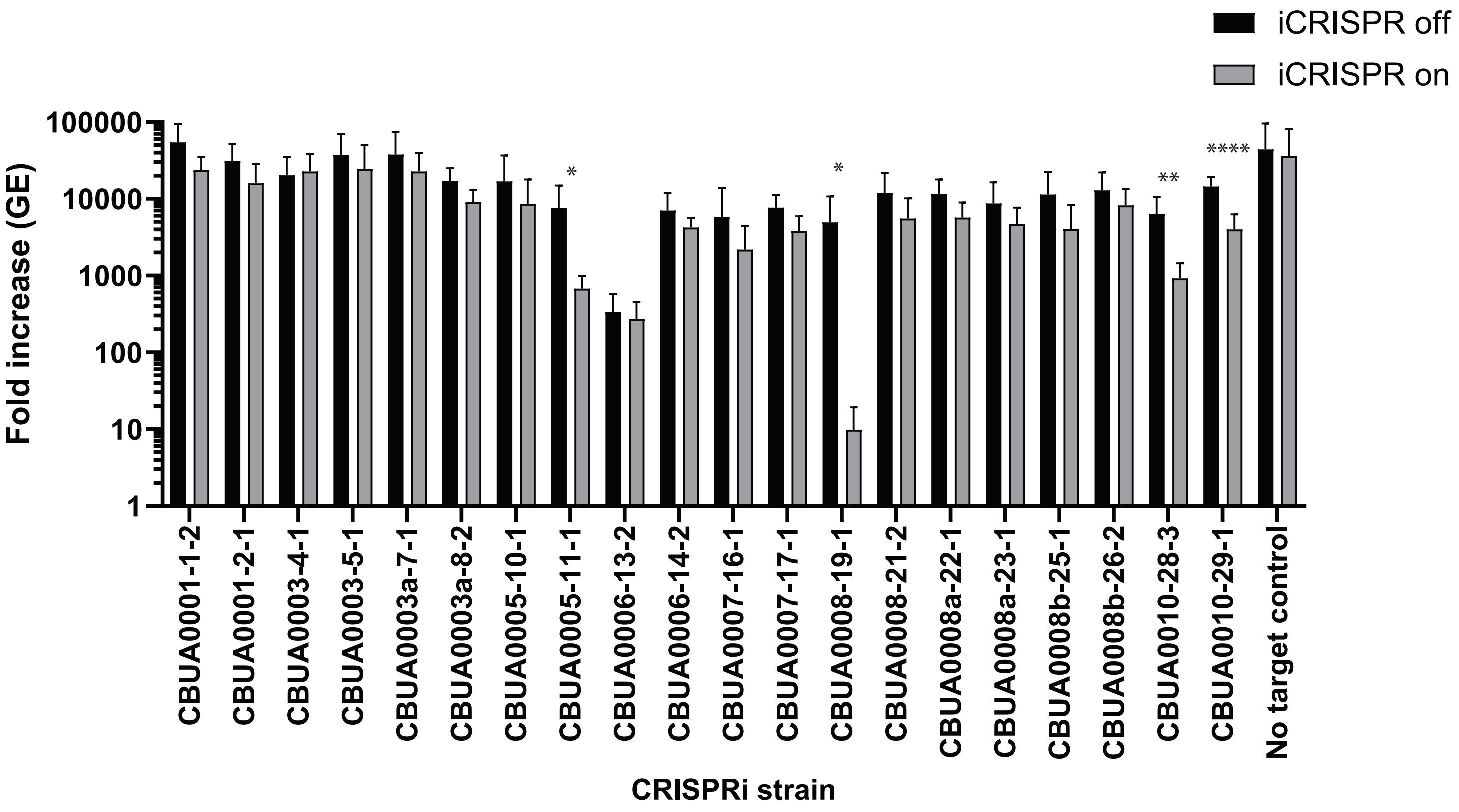

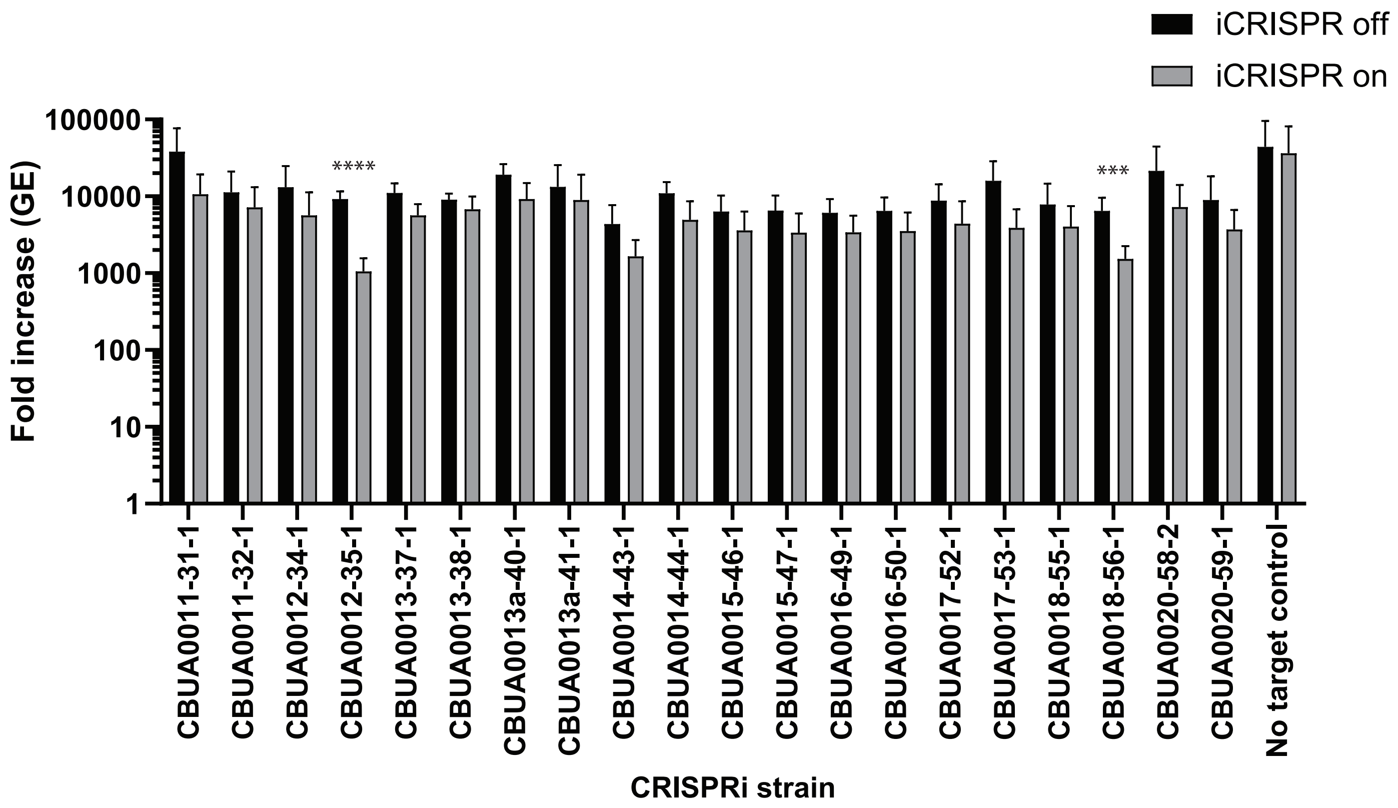

Supplemental figure 3

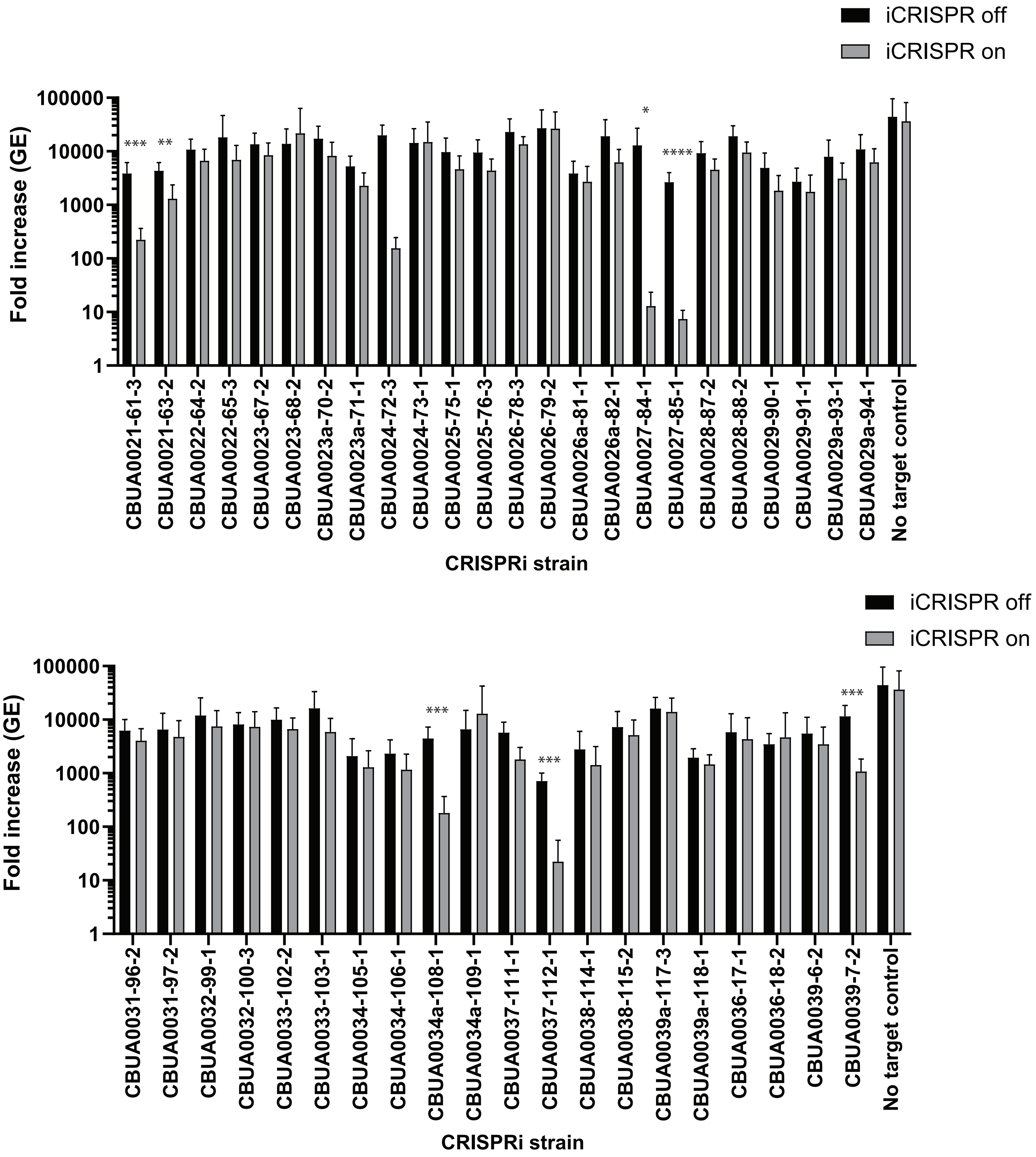

Supplemental figure 4

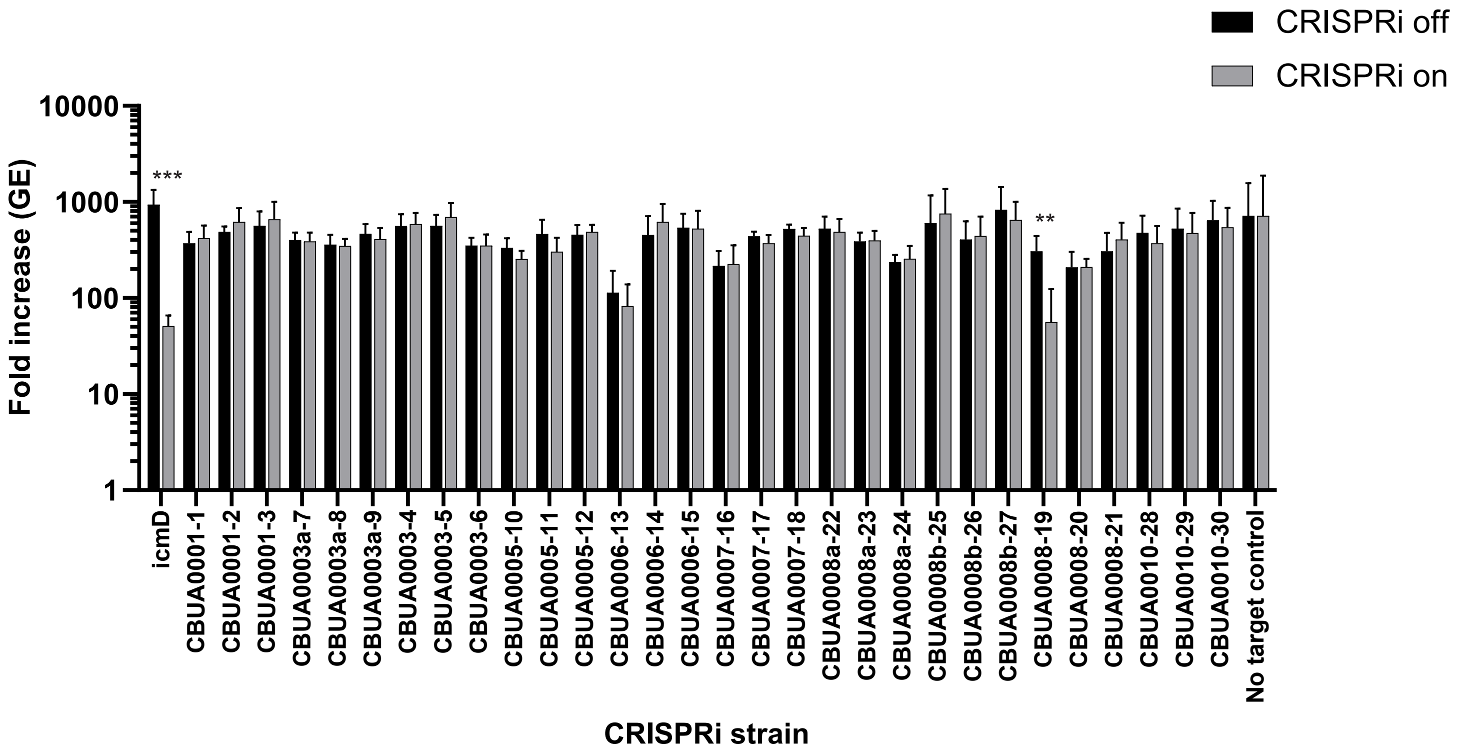

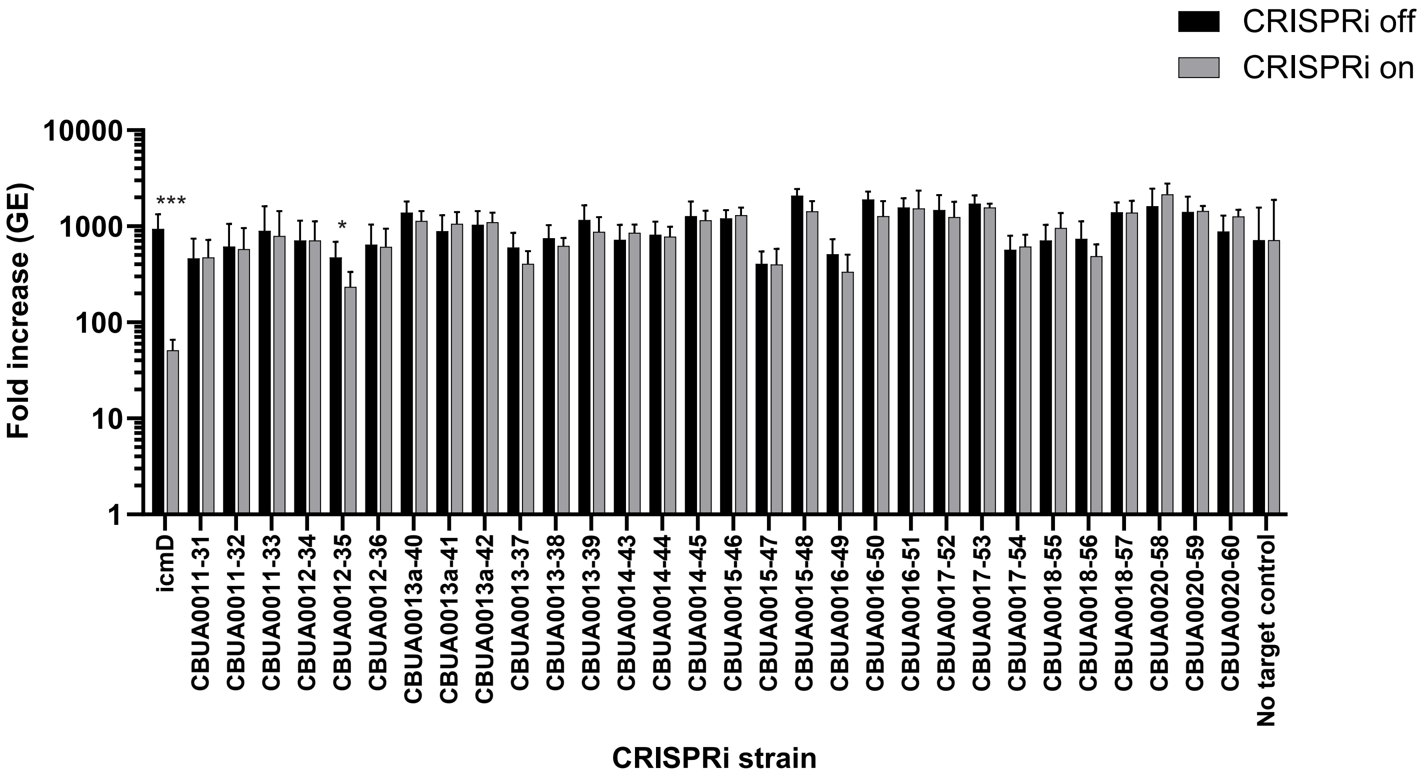

Supplemental figure 4

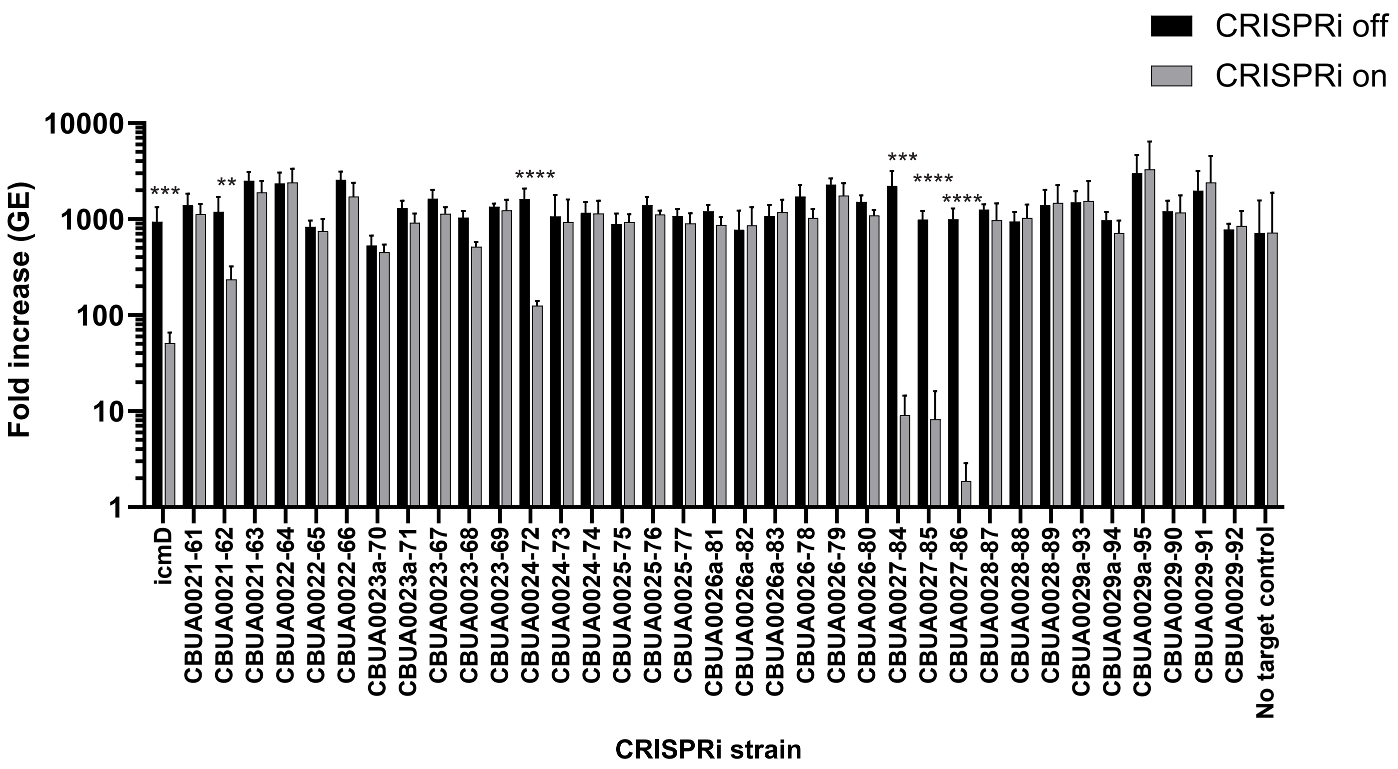

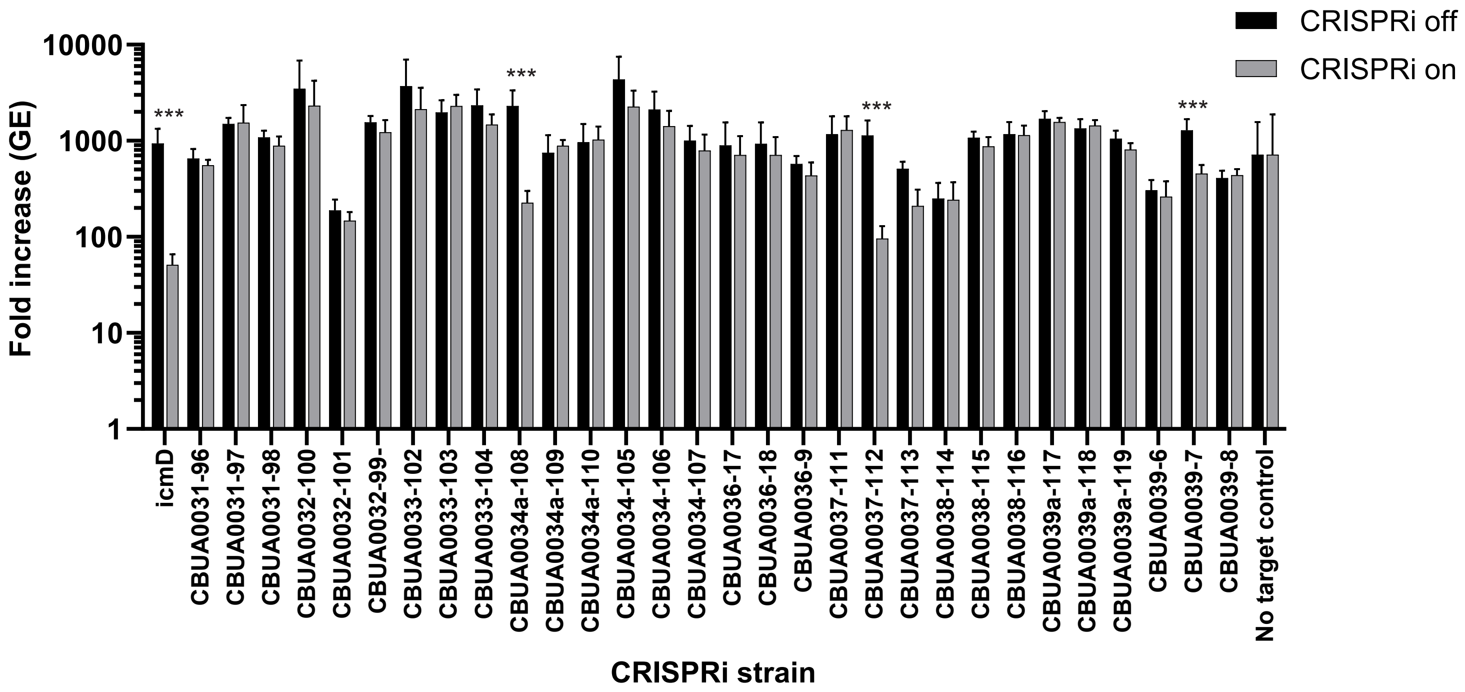

Supplemental figure 5

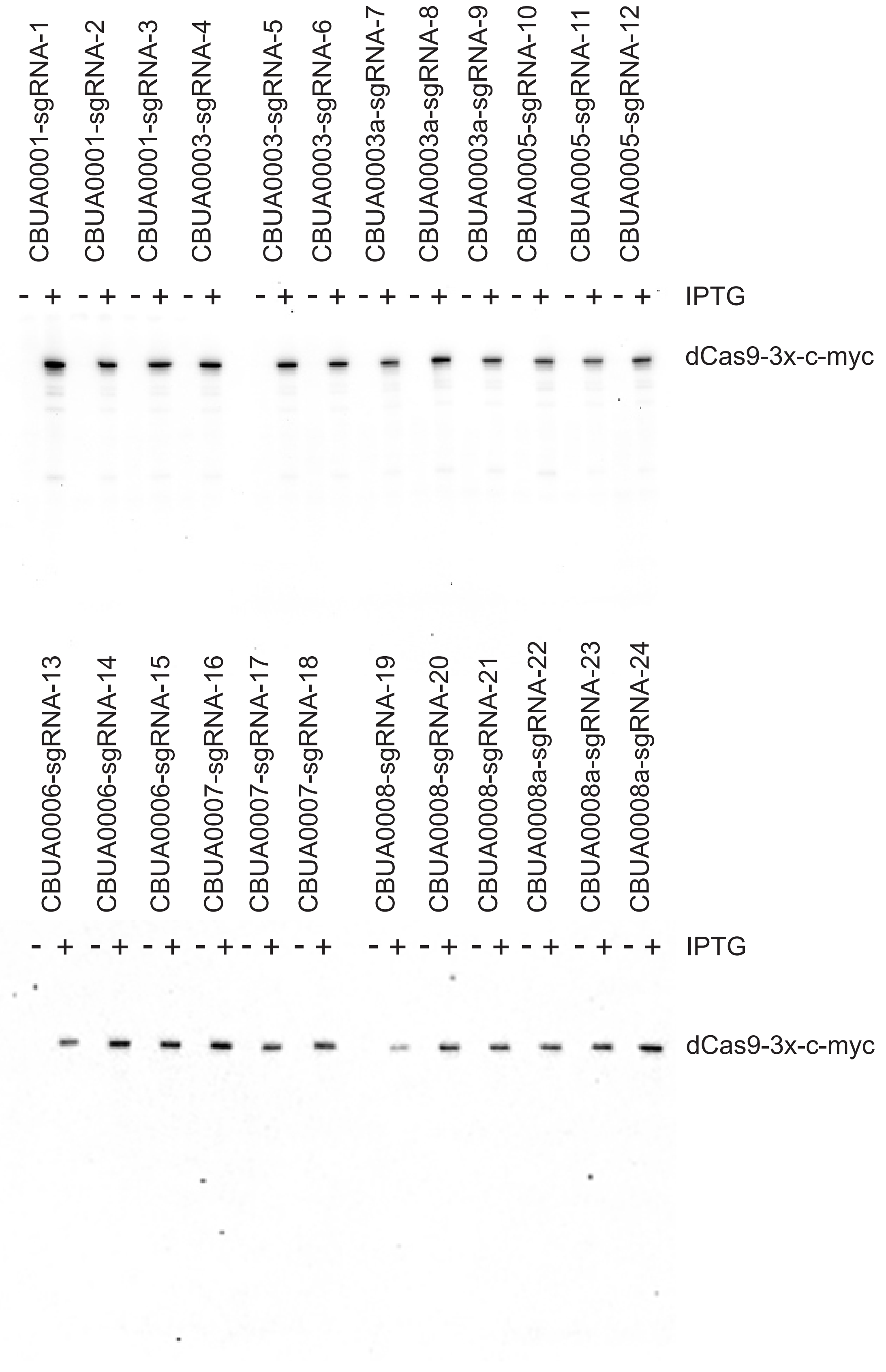

Supplemental figure 5

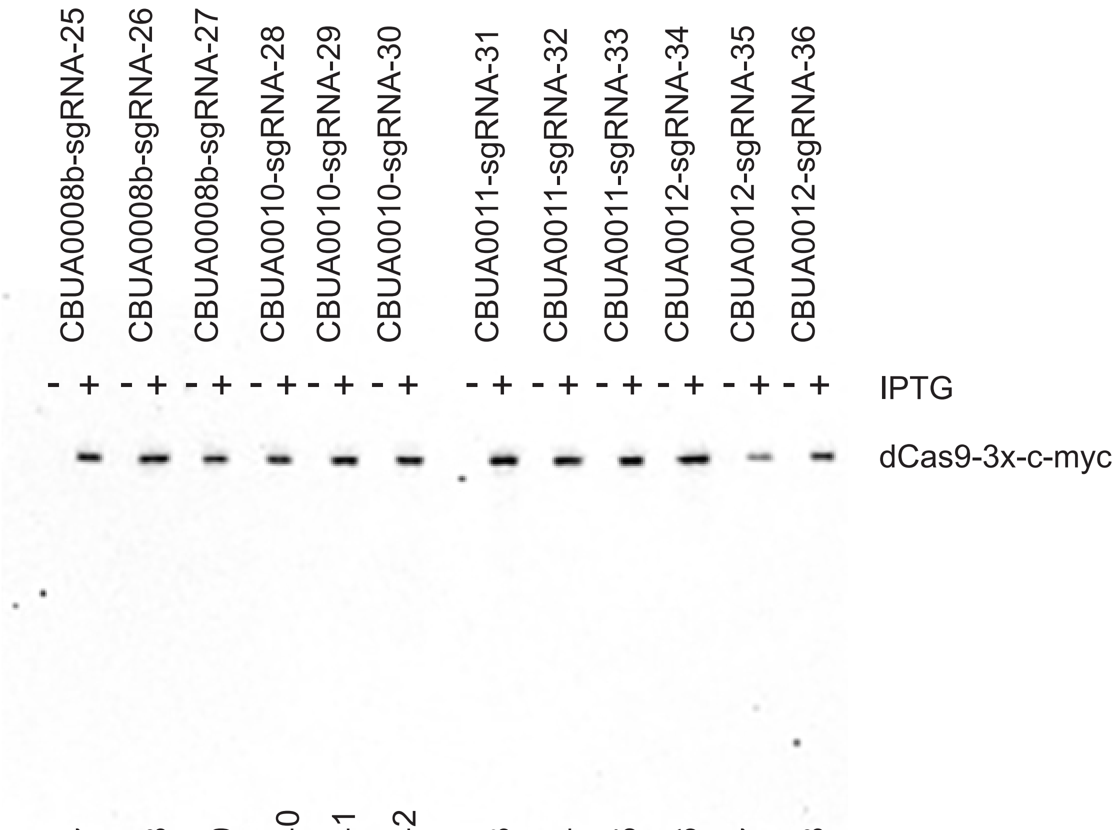

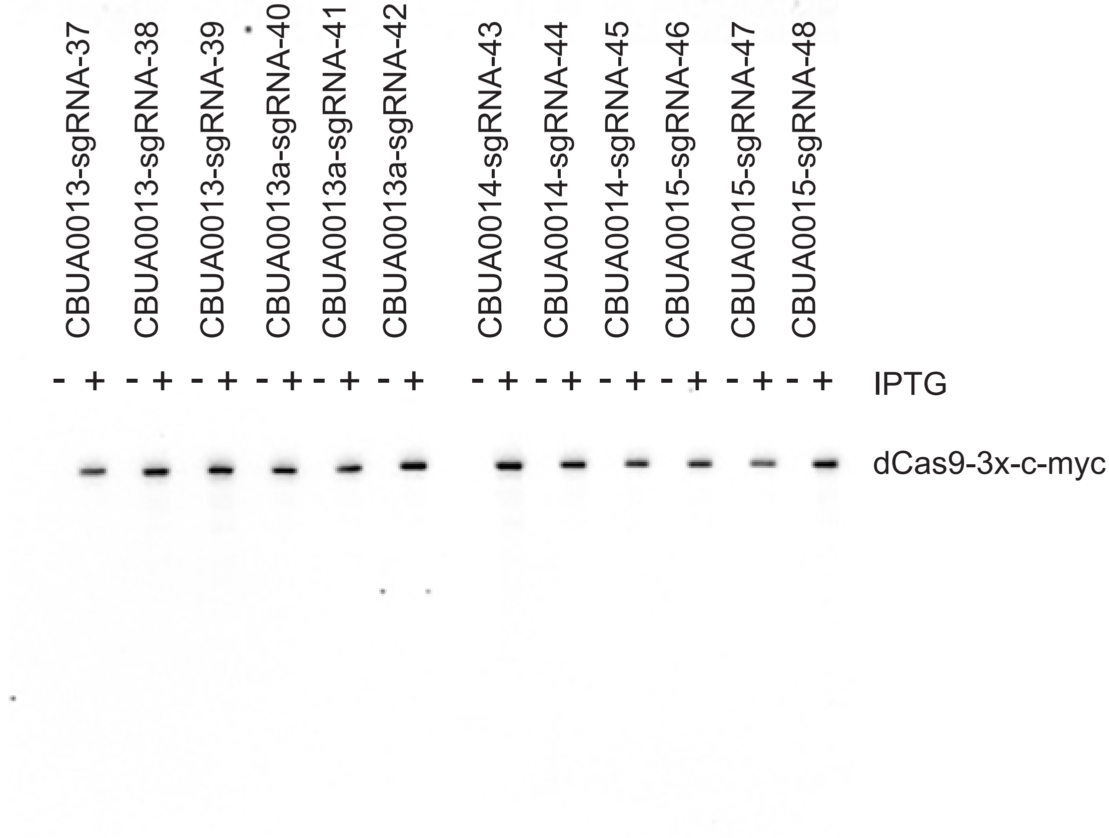

Supplemental figure 5

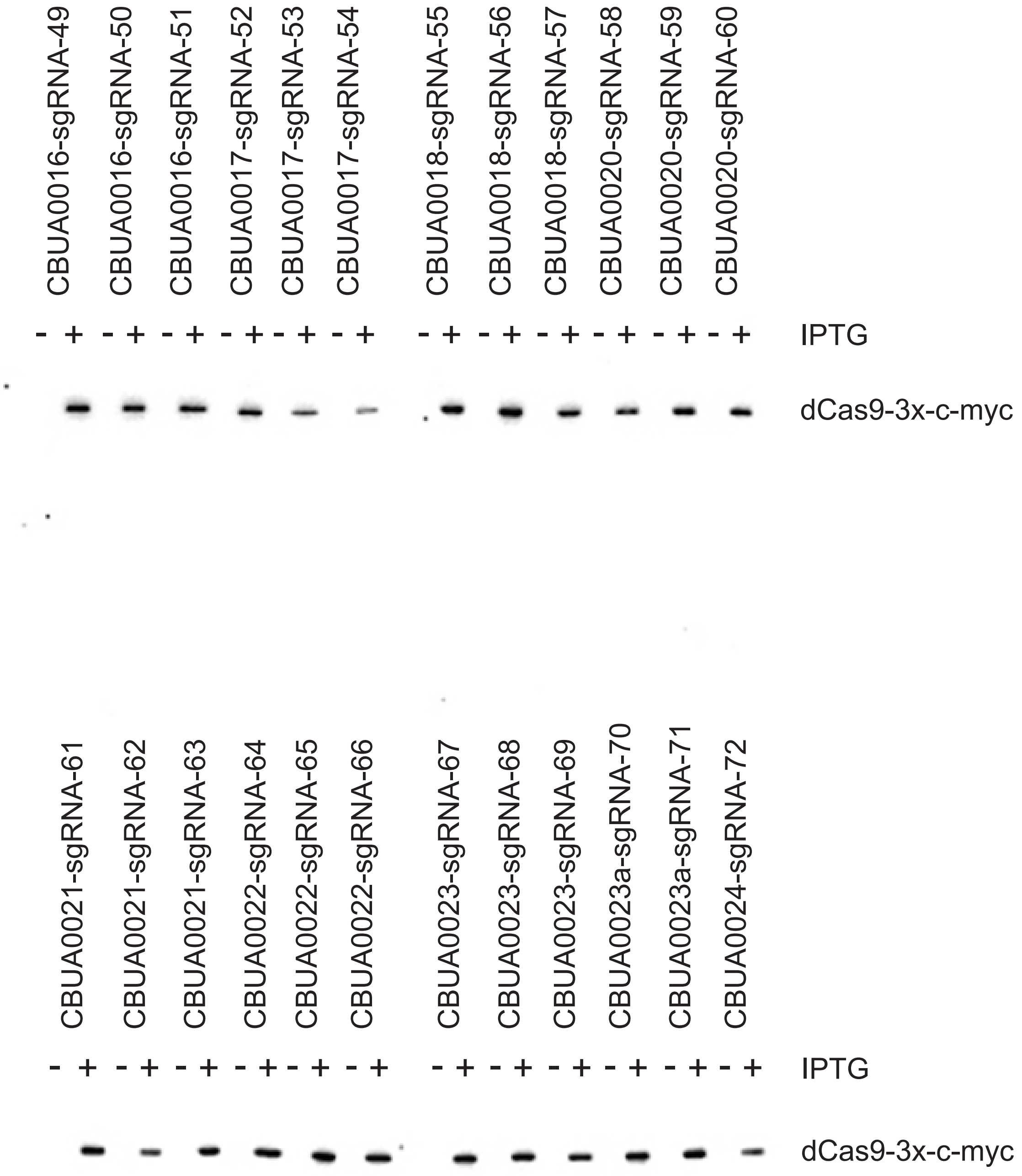

Supplemental figure 5

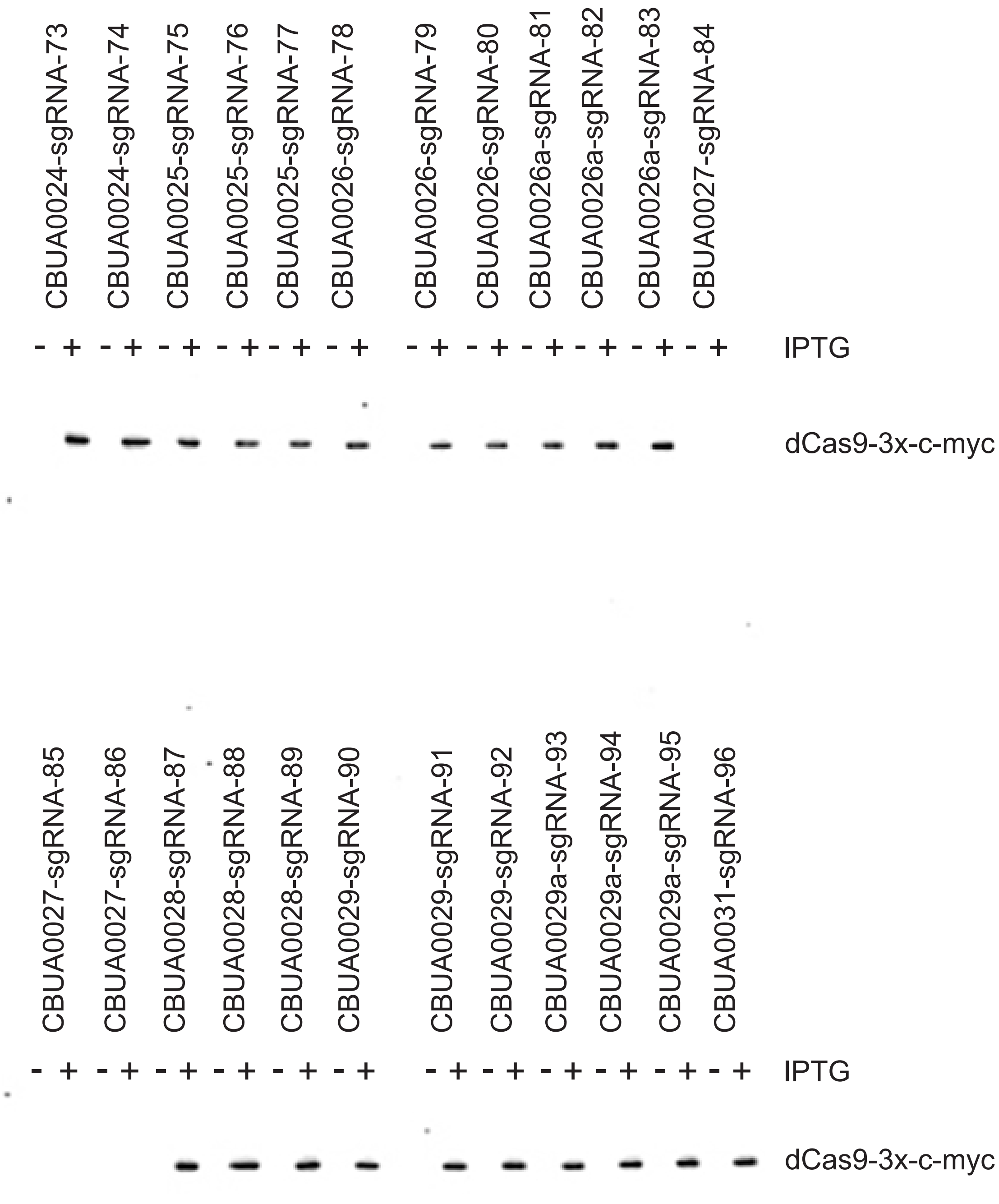

Supplemental figure 5

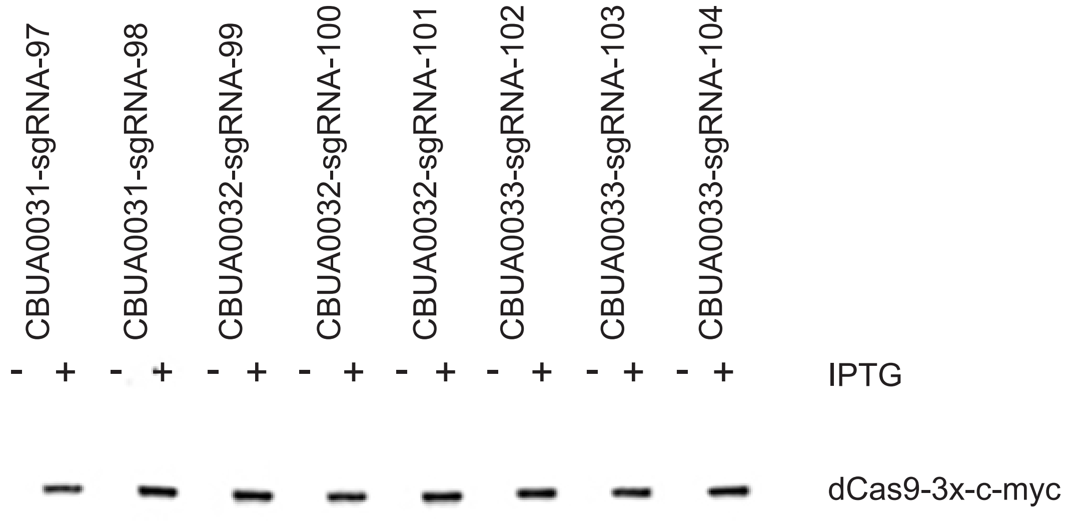

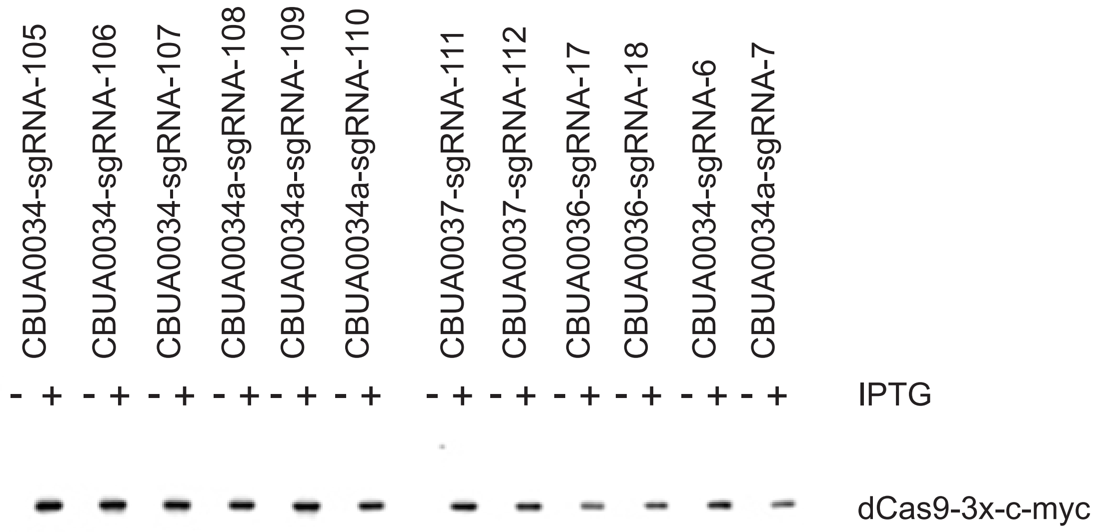

Supplemental figure 5

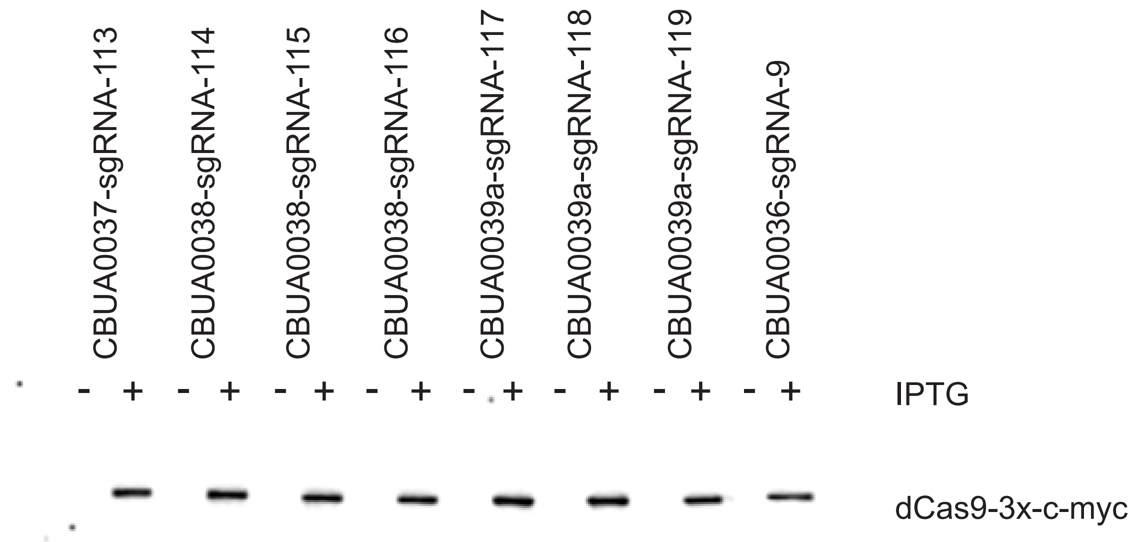

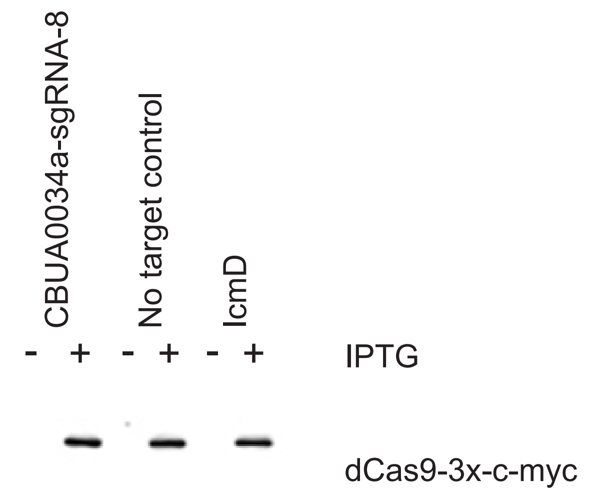

Supplemental figure 6

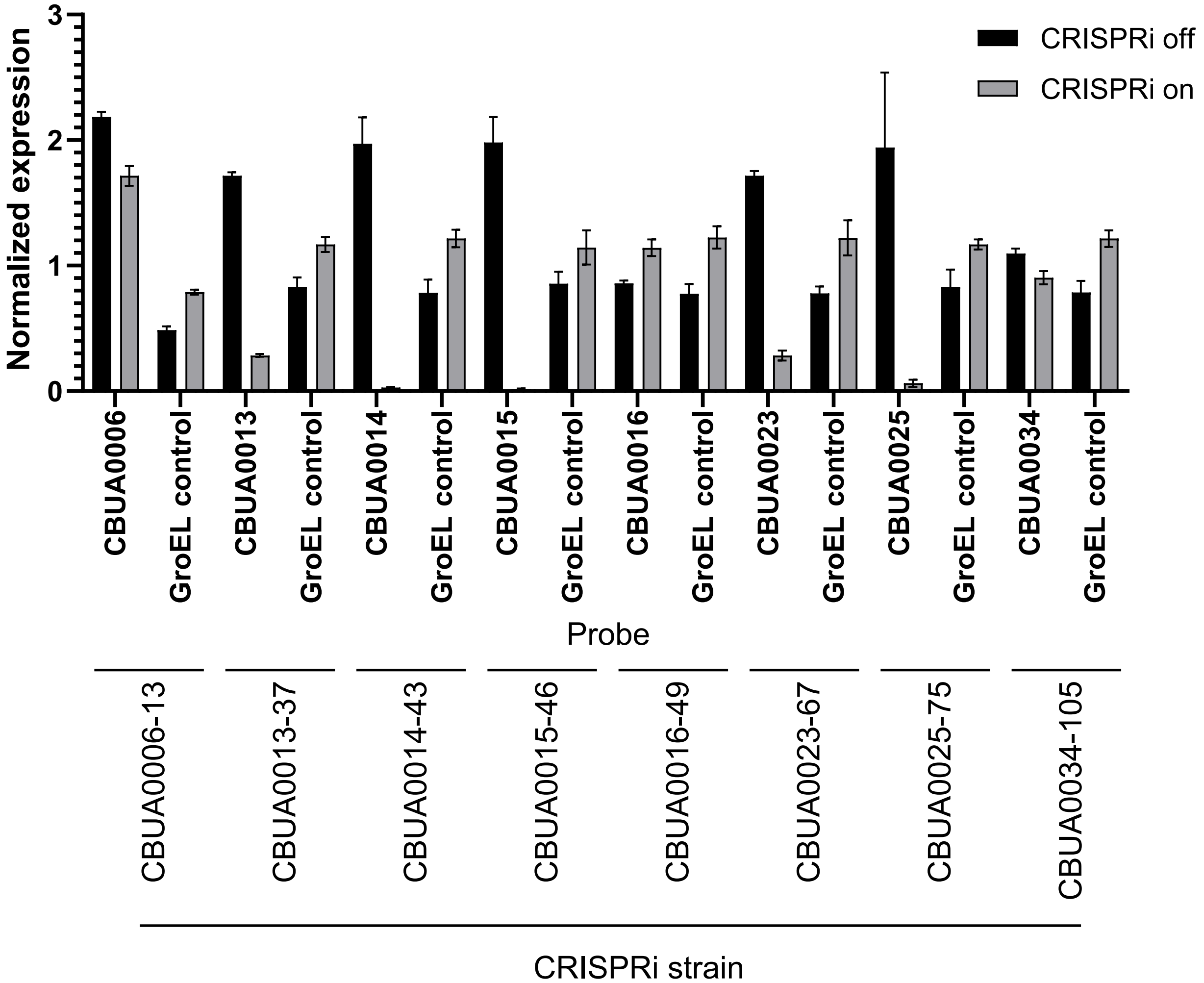

Supplemental figure 7

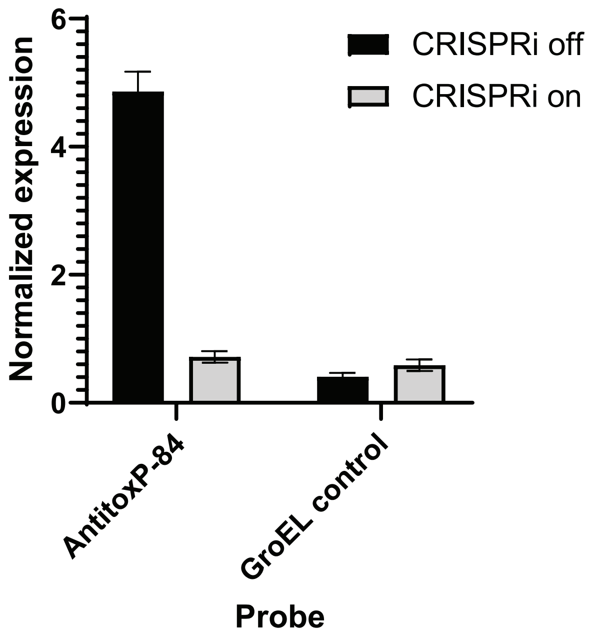

Supplemental figure 8

A

```
                                               -35
toxP    CTGATGGAAATCTGCTCGAGATAGGCAATGAGTACAGATTGACAAATAGTTCACTTAGTG 60
toxPG   ------------------------------------------------------------ 0

          -10
toxP    ATATAGTCTTAACAATAAGGGAAGTTATGAGGATATTTAAAACACGCTATTTCCATCGAT 120
toxPG   --AGTTTTTTGGAGAATAAATAACTAGGGCGCGTCGTCAATTAACCCTGCCACCGTCGTC 58
          *   * **    *  *   ** *   * *  *  * **   ** **    ** ***

toxP    GGGCAAAATCAGAAAATCTGTCCGATAGCCAACTGAAAACGGCCAT--TAGTGAACTGAT 178
toxPG   CCG----------CGGCTTGTCCGCGGGATCCAGAGAAACCACGTTAAGTTCTAGAAATT 108
          *               ******   *        ****  *  *       *     *

toxP    GAAGGGATTACACGATGGAGATTTAGGTTCCTATATCTATAAAAAACGGATTCCTATACT 238
toxPG   GGCTAGTTAATTAAGTATTCTGGGATATTCCTTTATCTATAAAAAACGGATTCCTATACT 168
        *    * * *     *        *  ***** ***************************

toxP    AGGTAAAGGAAAACGAGGTGGGCTTCGAACAATAATTGCTTACCGAGCAGAAGATAAAGC 298
toxPG   AGGTAAAGGAAAACGAGGTGGGCTTCGAACAATAATTGCTTACCGAGCAGAAGATAAAGC 228
        ************************************************************

toxP    TTTTTTTGTATATGGTTATGCGAAAAATGTTCAAGCCAATATAACTCCAAAAGAAAAGGA 358
toxPG   TTTTTTTGTATATGGTTATGCGAAAAATGTTCAAGCCAATATAACTCCAAAAGAAAAGGA 288
        ************************************************************

toxP    GGCGTACAAAAAGTTGTCAAAAACTTATTTTGATATGAAAGAATTAGAGCTTCAATCATT 418
toxPG   GGCGTACAAAAAGTTGTCAAAAACTTATTTTGATATGAAAGAATTAGAGCTTCAATCATT 348
        ************************************************************

toxP    ATTAAAAATAGGCGAACTGATAGAGGTGCTGTGA 452
toxPG   ATTAAAAATAGGCGAACTGATAGAGGTGCTGTGA 382
        **********************************
```

B

```
antitoxP   ATGAAAGATAAAAAAAAGAAAAGTAAAACTCGTTTTAAACCTTTATCGCCCATTGAGGCC 60
antitoxPG  ATGAAAGATAAAAAAAAGAAAAGTAAAACTCGTTTTAAACCTTTATCGCCCATTGAGGCC 60
           ************************************************************

antitoxP   GTTCATGAATCTGCAAAAGGATTATATGATGCCGGTGTTATTGACGCGACAACAATGCAT 120
antitoxPG  GTTCATGAATCTGCAAAAGGATTATATGATGCCGGTGTTATTGACGCGACAACAATGCAT 120
           ************************************************************

antitoxP   GAATTTGATGCATTGTGTTTGACTCCAGTCCGTGAATTATCGCCTCGTGAAATTAAGCGC 180
antitoxPG  GAATTTGATGCATTGTGTTTGACTCCAGTCCGTGAATTATCGCCTCGTGAAATTAAGCGC 180
           ************************************************************

antitoxP   ATTCGAATTCATGAAAAAGTGAGCCAGGCCGTTTTTGCAAAATATTTGAATACCAGTGTT 240
antitoxPG  ATTCGAATTCATGAAAAAGTGAGCCAGGCCGTTTTTGCAAAATATTTGAATACCAGTGTT 240
           ************************************************************

antitoxP   TCTACGGTAAAGCAATGGGAGTTGGGTGAGAAACACCCTCGAGGCACTTCATTAAAATTA 300
antitoxPG  TCTACGGTAAAGCAATGGGAGTTGGGTGAGAAACATCCTCGAGGCACTTCATTAAAATTA 300
           *********************************** ************************

antitoxP   CTGAACCTCGTTGATAGAAAAGGCCTTCAAGCCATCGCTTAG 342
antitoxPG  CTGAACCTCGTTGATAGAAAAGGCCTTCAAGCCATCGCTTAG 342
           ******************************************
```

Supplemental figure 9

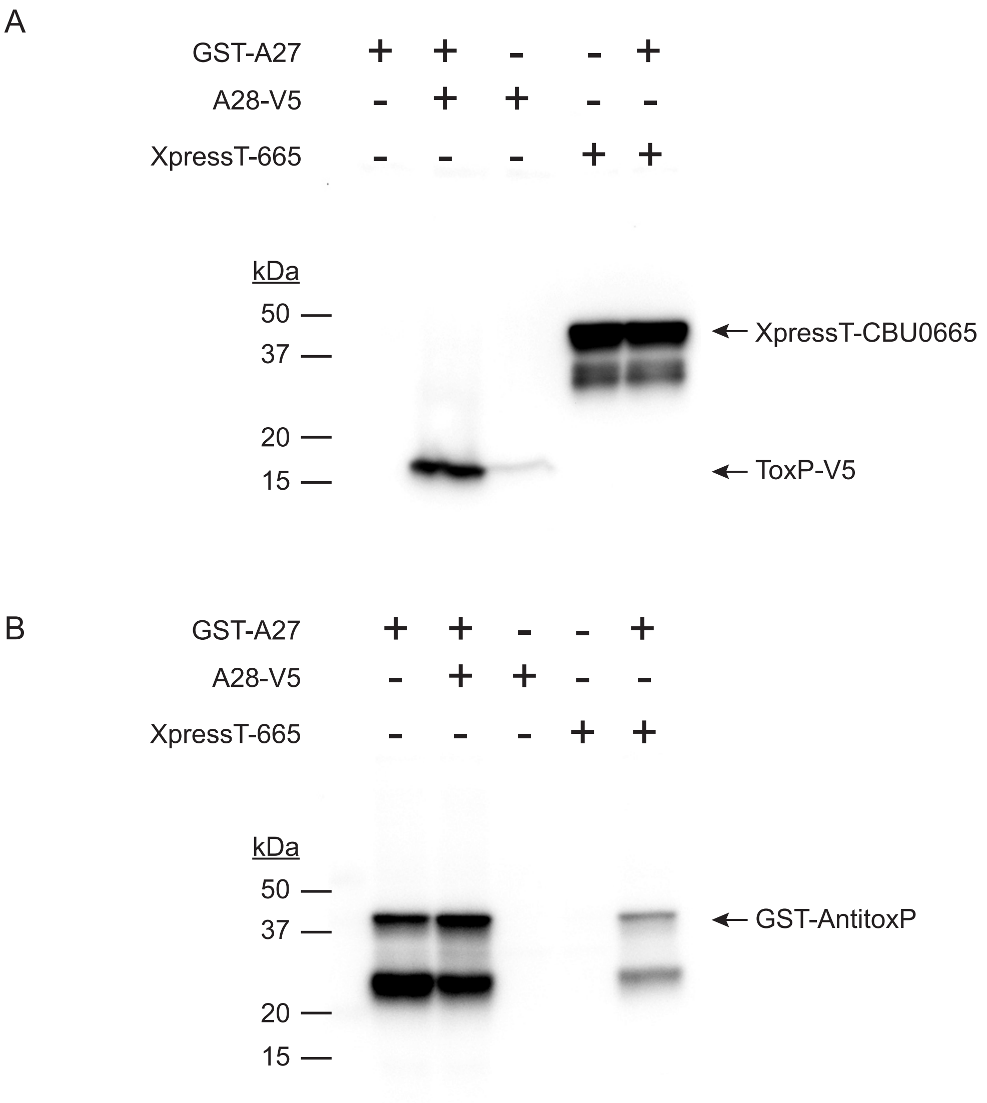

Supplemental figure 10

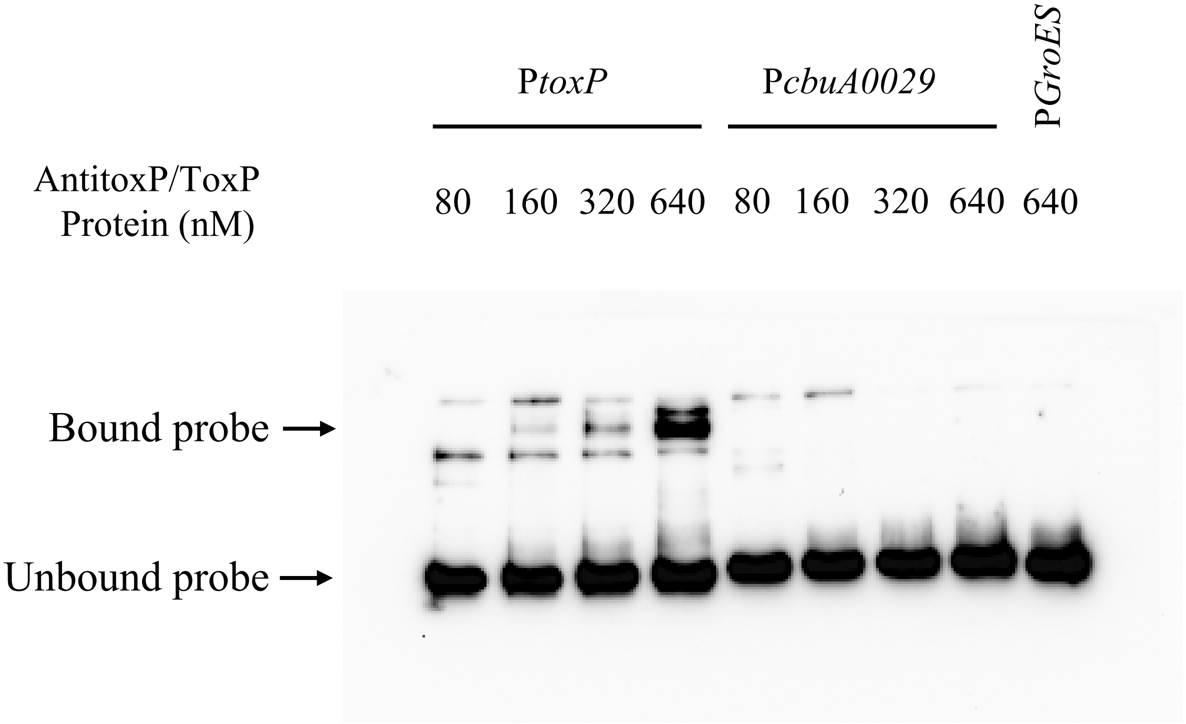

Supplemental figure 11

```
                                                          -35
antitoxP/toxP       CTGATGGAAATCTGCTCGAGATAGGCAATGAGTACAGATTGACAAATAGTTCACTTAGTG 60
toxPG/antitoxPG     ------------------------------------------------------------ 0

                      -10
antitoxP/toxP       ATATAGTCTTAACAATAAGGGAAGTTATGAGGATATTT-AAA------AC---------- 103
toxPG/antitoxPG     ----------------------AGTTTTTTGGAGAATAAATAACTAGGGCGCGTCGTCAA 38
                                          ****:* :*** *:*: *:*      .*

antitoxP/toxP       ---ACGCTATTTCCATCGATGGGCAAAATCAGAAAATCTGTCCGATA--GCCAACTGAAA 158
toxPG/antitoxPG     TTAACCCTGCCACCGTCGTCCCGCG------G----CTTGTCCGCGGATCCAGA-G-AA 86
                       ** **.  :**.***:   **.      *      ******. .   ***.. * **
                                                         -35
antitoxP/toxP       ACGGCCATTAGTGAACTGATGAAGGGATTACAC-GATGGAGA---TTTAGGTTCCTATAT 214
toxPG/antitoxPG     ACCACGTT--AAGTTCTAGAAATTGGCTAGTTAATTAAGTATTCTGGGATATTCCTTTAT 144
                    ** .* :*  .:*::**..:.*: **.*:. :.  ::.*:.:      * .*****:***
                      -10
antitoxP/toxP       CTATAAAAAACGGATTCCTATACTAGGTAAAGGAAAACGAGGTGGGCTTCGAACAATAAT 274
toxPG/antitoxPG     CTATAAAAAACGGATTCCTATACTAGGTAAAGGAAAACGAGGTGGGCTTCGAACAATAAT 204
                    ************************************************************

antitoxP/toxP       TGCTTACCGAGCAGAAGATAAAGCTTTTTTTGTATATGGTTATGCGAAAAATGTTCAAGC 334
toxPG/antitoxPG     TGCTTACCGAGCAGAAGATAAAGCTTTTTTTGTATATGGTTATGCGAAAAATGTTCAAGC 264
                    ************************************************************
                                                                          -35
antitoxP/toxP       CAATATAACTCCAAAAGAAAAGGAGGCGTACAAAAAGTTGTCAAAAACTTATTTTGATAT 394
toxPG/antitoxPG     CAATATAACTCCAAAAGAAAAGGAGGCGTACAAAAAGTTGTCAAAAACTTATTTTGATAT 324
                    ************************************************************
                                   -10
antitoxP/toxP       GAAAGAATTAGAGCTTCAATCATTATTAAAAATAGGCGAACTGATAGAGGTGCTGTGATG 454
toxPG/antitoxPG     GAAAGAATTAGAGCTTCAATCATTATTAAAAATAGGCGAACTGATAGAGGTGCTGTGATG 384
                    ************************************************************

antitoxP/toxP       AAAGATAAAAAAAAGAAAAGTAAAACTCGTTTTAAACCTTTATCGCCCATTGAGGCCGTT 514
toxPG/antitoxPG     AAAGATAAAAAAAAGAAAAGTAAAACTCGTTTTAAACCTTTATCGCCCATTGAGGCCGTT 444
                    ************************************************************

antitoxP/toxP       CATGAATCTGCAAAAGGATTATATGATGCCGGTGTTATTGACGCGACAACAATGCATGAA 574
toxPG/antitoxPG     CATGAATCTGCAAAAGGATTATATGATGCCGGTGTTATTGACGCGACAACAATGCATGAA 504
                    ************************************************************

antitoxP/toxP       TTTGATGCATTGTGTTTGACTCCAGTCCGTGAATTATCGCCTCGTGAAATTAAGCGCATT 634
toxPG/antitoxPG     TTTGATGCATTGTGTTTGACTCCAGTCCGTGAATTATCGCCTCGTGAAATTAAGCGCATT 564
                    ************************************************************

antitoxP/toxP       CGAATTCATGAAAAAGTGAGCCAGGCCGTTTTTGCAAAATATTTGAATACCAGTGTTTCT 694
toxPG/antitoxPG     CGAATTCATGAAAAAGTGAGCCAGGCCGTTTTTGCAAAATATTTGAATACCAGTGTTTCT 624
                    ************************************************************

antitoxP/toxP       ACGGTAAAGCAATGGGAGTTGGGTGAGAAACACCCTCGAGGCACTTCATTAAAATTACTG 754
toxPG/antitoxPG     ACGGTAAAGCAATGGGAGTTGGGTGAGAAACATCCTCGAGGCACTTCATTAAAATTACTG 684
                    ******************************** ***************************

antitoxP/toxP       AACCTCGTTGATAGAAAAGGCCTTCAAGCCATCGCTTAG                      793
toxPG/antitoxPG     AACCTCGTTGATAGAAAAGGCCTTCAAGCCATCGCTTAG                      723
                    ***************************************
```

Supplemental figure 12

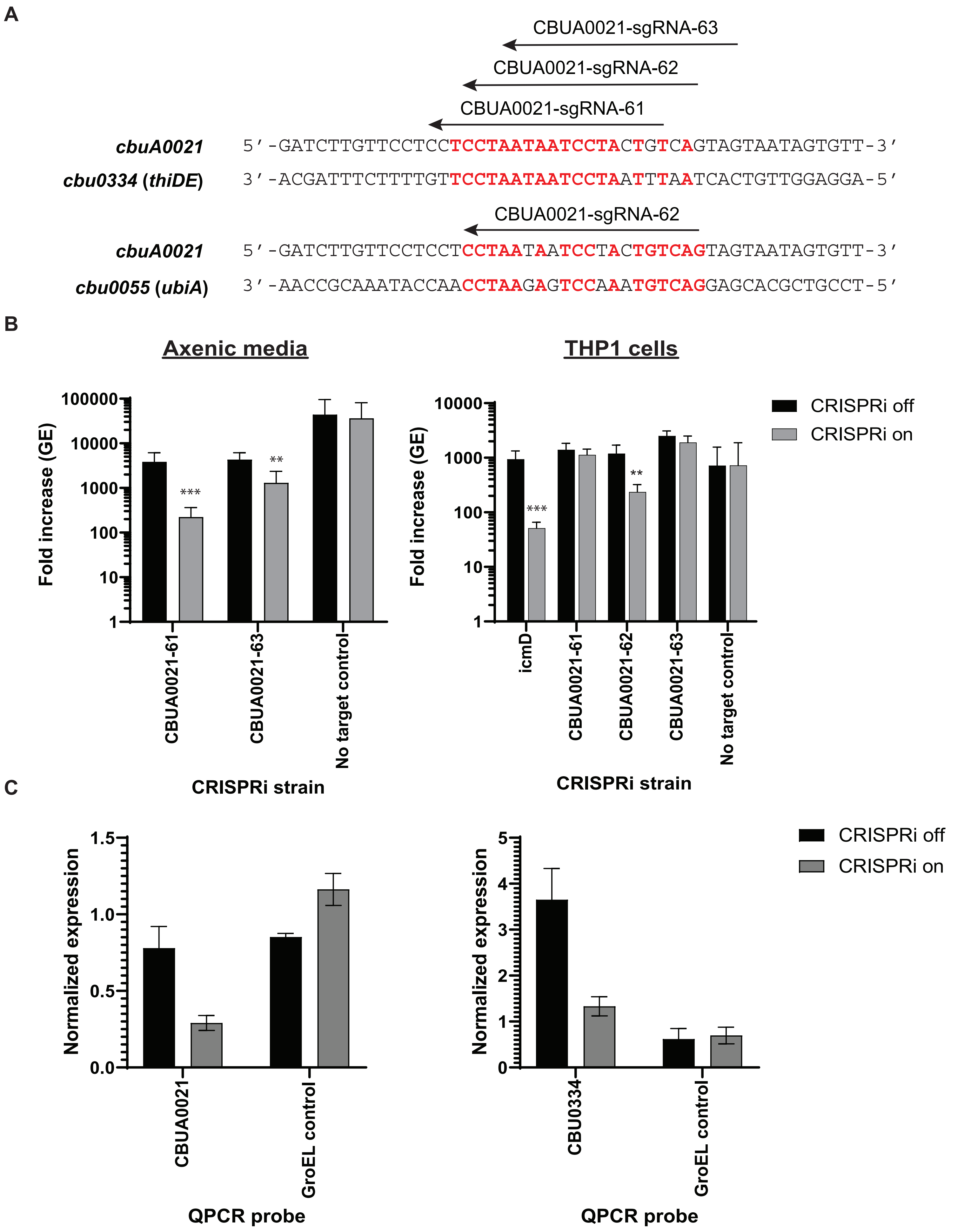

Supplemental figure 13

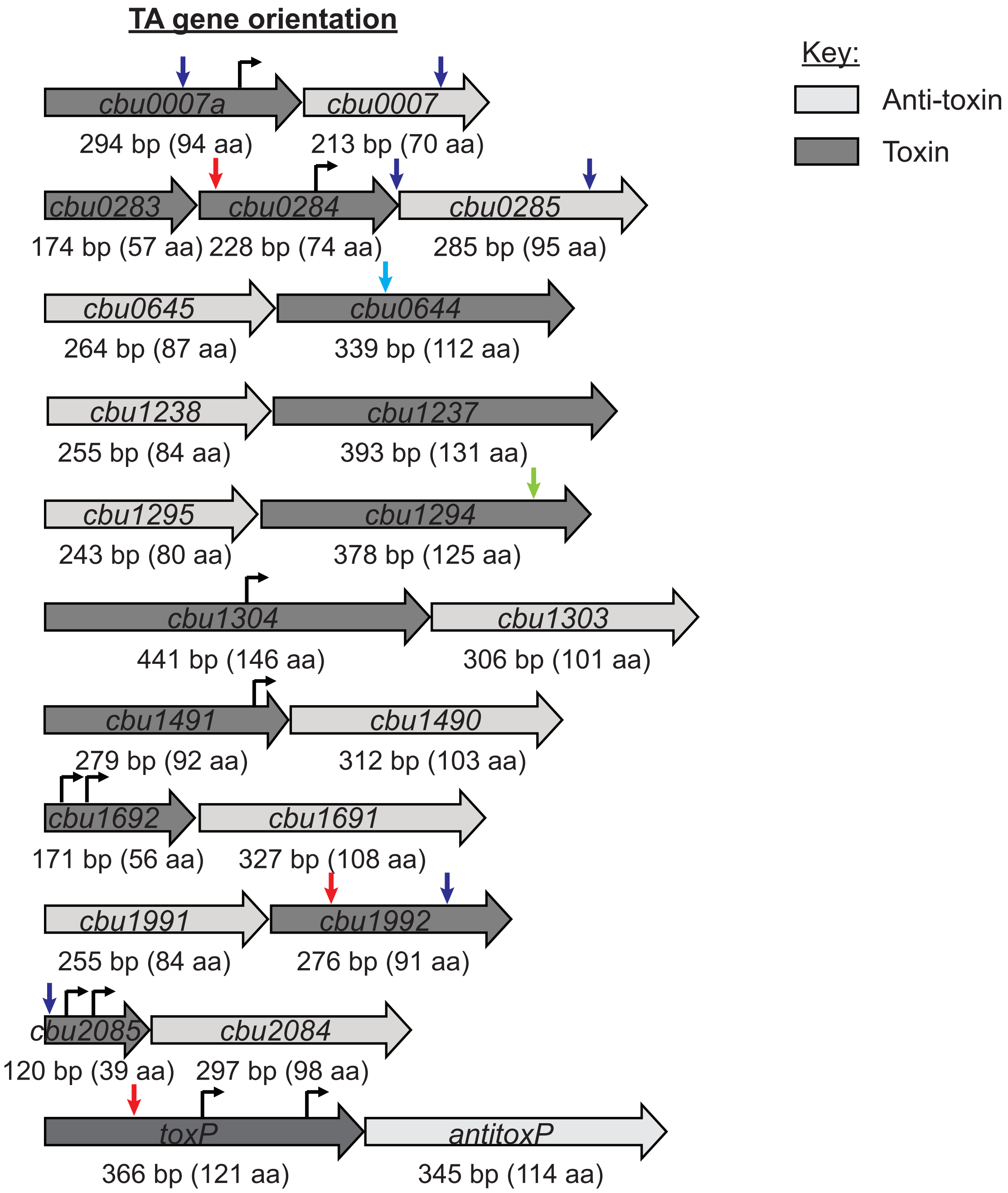

**Strain or plasmid**	**Genotype and/or phenotype**	**Source/reference**
Strain		
E. coli Stellar cells	*F–, endA1, supE44, thi-1, recA1, relA1, gyrA96, phoA, Φ80D lacZΔ M15, Δ (lacZYA - argF) U169, Δ(mrr - hsdRMS - mcrBC), ΔmcrA, λ–*	Takara
E. coli PIR1 cells	*F- Δlac169, rpoS(Am), robA1, creC510, hsdR514, endA, recA1, uidA(ΔMluI)::pir-116*	Invitrogen
C. burnetii Nine Mile RSA439 (NMII)	Phase II, clone 4	Beare *et_al* 2012
NMII ΔQpH1	NMII QpH1-less strain containing pB-TyrB-QpH1ori	This study
Plasmids		
QpH1	Endogenous *C. burnetii* plasmid	This study
pTnS2::*1169*^{P}-*tnsABCD*	*cbu1169* promoter cloned into pTnS2, R6K *ori*, Amp^r	Beare *et_al* 2011
pJB-CAT-LacIO-MC	*lacIO* cloned into pJB-CAT, replacing *lacIq*-P*tac*, Amp^r, Cm^r	This study
Coxiella shuttle vector		
pJB-CAT	pJB2581-*mCherry* containing cat driven by *1169*^{P}; Amp^r, Cm^r	Omsland *et_al* 2011
pJB-CAT-*tyrB*	pJB-CAT containing *tyrB* driven by *1169*^{P}, Cmr, Amp^r	This study
pJC-CAT	pJC84 containing *cat* driven by *1169*^{P}; Cm^r	Beare *et_al* 2011
pB-TyrB	*cbu1169* promoter driving expression of *cat* and *sacB*, pMB1 *ori*, Cm^r	This study
pB-TyrB-QpH1ori	*cbuA0036-cbuA0037-cbuA0038-cbuA0039* cloned into pB-TyrB, Cm^r	This study
CRISPRi constructs		
pJMP1356	pMiniTn7 vector containing elements of a CRISPRi system, dCas9, lacI, and targeting sgRNA, R6k *ori*, Amp^r, Cm^r	Peters *et_al* 2019
pB-iCRISPR	*proBA* cloned in place of cat in pJMP1356, R6K *ori*, Amp^r	This study
pB-iCRISPR-*icmD*-sgRNA-1	pB-iCRISPR containing *cbu1624*-sgRNA-1 target sequence, R6K *ori*, Amp^r	This study
pB-iCRISPR-*icmD*-sgRNA-2	pB-iCRISPR containing *cbu1624*-sgRNA-2 target sequence, R6K *ori*, Amp^r	This study
pB-iCRISPR-*icmD*-sgRNA-1-*scvA*-sgRNA-1	pB-iCRISPR containing *cbu1624*-sgRNA-1 (*icmD*) and *cbu1267a* (*scvA*) target sequences, R6K *ori*, Amp^r	This study
pB-iCRISPR-*icmD*-sgRNA-3	pB-iCRISPR containing *cbu1624*-sgRNA-3 target sequence, R6K *ori*, Amp^r	This study
pB-iCRISPR-*cbuA0001*-sgRNA-1	pB-iCRISPR containing *cbuA0001*-sgRNA-1 target sequence, R6K *ori*, Amp^r	This study
pB-iCRISPR-*cbuA0001*-sgRNA-2	pB-iCRISPR containing *cbuA0001*-sgRNA-2 target sequence, R6K *ori*, Amp^r	This study
pB-iCRISPR-*cbuA0001*-sgRNA-3	pB-iCRISPR containing *cbuA0001*-sgRNA-3 target sequence, R6K *ori*, Amp^r	This study
pB-iCRISPR-*cbuA0003*-sgRNA-4	pB-iCRISPR containing *cbuA0003*-sgRNA-4 target sequence, R6K *ori*, Amp^r	This study
pB-iCRISPR-*cbuA0003*-sgRNA-5	pB-iCRISPR containing *cbuA0003*-sgRNA-5 target sequence, R6K *ori*, Amp^r	This study
pB-iCRISPR-*cbuA0003*-sgRNA-6	pB-iCRISPR containing *cbuA0003*-sgRNA-6 target sequence, R6K *ori*, Amp^r	This study
pB-iCRISPR-*cbuA0003a*-sgRNA-7	pB-iCRISPR containing *cbuA0003a*-sgRNA-7 target sequence, R6K *ori*, Amp^r	This study
pB-iCRISPR-*cbuA0003a*-sgRNA-8	pB-iCRISPR containing *cbuA0003a*-sgRNA-8 target sequence, R6K *ori*, Amp^r	This study
pB-iCRISPR-*cbuA0003a*-sgRNA-9	pB-iCRISPR containing *cbuA0003a*-sgRNA-9 target sequence, R6K *ori*, Amp^r	This study
pB-iCRISPR-*cbuA0005*-sgRNA-10	pB-iCRISPR containing *cbuA0005*-sgRNA-10 target sequence, R6K *ori*, Amp^r	This study
pB-iCRISPR-*cbuA0005*-sgRNA-11	pB-iCRISPR containing *cbuA0005*-sgRNA-11 target sequence, R6K *ori*, Amp^r	This study
pB-iCRISPR-*cbuA0005*-sgRNA-12	pB-iCRISPR containing *cbuA0005*-sgRNA-12 target sequence, R6K *ori*, Amp^r	This study
pB-iCRISPR-*cbuA0006*-sgRNA-13	pB-iCRISPR containing *cbuA0006*-sgRNA-13 target sequence, R6K *ori*, Amp^r	This study
pB-iCRISPR-*cbuA0006*-sgRNA-14	pB-iCRISPR containing *cbuA0006*-sgRNA-14 target sequence, R6K *ori*, Amp^r	This study
pB-iCRISPR-*cbuA0006*-sgRNA-15	pB-iCRISPR containing *cbuA0006*-sgRNA-15 target sequence, R6K *ori*, Amp^r	This study
pB-iCRISPR-*cbuA0007*-sgRNA-16	pB-iCRISPR containing *cbuA0007*-sgRNA-16 target sequence, R6K *ori*, Amp^r	This study
pB-iCRISPR-*cbuA0007*-sgRNA-17	pB-iCRISPR containing *cbuA0007*-sgRNA-17 target sequence, R6K *ori*, Amp^r	This study
pB-iCRISPR-*cbuA0007*-sgRNA-18	pB-iCRISPR containing *cbuA0007*-sgRNA-18 target sequence, R6K *ori*, Amp^r	This study
pB-iCRISPR-*cbuA0008*-sgRNA-19	pB-iCRISPR containing *cbuA0008*-sgRNA-19 target sequence, R6K *ori*, Amp^r	This study
pB-iCRISPR-*cbuA0008*-sgRNA-20	pB-iCRISPR containing *cbuA0008*-sgRNA-20 target sequence, R6K *ori*, Amp^r	This study
pB-iCRISPR-*cbuA0008*-sgRNA-21	pB-iCRISPR containing *cbuA0008*-sgRNA-21 target sequence, R6K *ori*, Amp^r	This study
pB-iCRISPR-*cbuA0008a*-sgRNA-22	pB-iCRISPR containing *cbuA0008a*-sgRNA-22 target sequence, R6K *ori*, Amp^r	This study
pB-iCRISPR-*cbuA000*8a-sgRNA-23	pB-iCRISPR containing *cbuA0008a*-sgRNA-23 target sequence, R6K *ori*, Amp^r	This study
pB-iCRISPR-*cbuA0008a*-sgRNA-24	pB-iCRISPR containing *cbuA0008a*-sgRNA-24 target sequence, R6K *ori*, Amp^r	This study

pB-iCRISPR-*cbuA0008b* -sgRNA-25	pB-iCRISPR containing *cbuA0008b* -sgRNA-25 target sequence, R6K *ori* , Amp^r	This study
pB-iCRISPR-*cbuA0008b* -sgRNA-26	pB-iCRISPR containing *cbuA0008b* -sgRNA-26 target sequence, R6K *ori* , Amp^r	This study
pB-iCRISPR-*cbuA0008b* -sgRNA-27	pB-iCRISPR containing *cbuA0008b* -sgRNA-27 target sequence, R6K *ori* , Amp^r	This study
pB-iCRISPR-*cbuA0010* -sgRNA-28	pB-iCRISPR containing *cbuA0010* -sgRNA-28 target sequence, R6K *ori* , Amp^r	This study
pB-iCRISPR-*cbuA0010* -sgRNA-29	pB-iCRISPR containing *cbuA0010* -sgRNA-29 target sequence, R6K *ori* , Amp^r	This study
pB-iCRISPR-*cbuA0010* -sgRNA-30	pB-iCRISPR containing *cbuA0010* -sgRNA-30 target sequence, R6K *ori* , Amp^r	This study
pB-iCRISPR-*cbuA0011* -sgRNA-31	pB-iCRISPR containing *cbuA0011* -sgRNA-31 target sequence, R6K *ori* , Amp^r	This study
pB-iCRISPR-*cbuA0011* -sgRNA-32	pB-iCRISPR containing *cbuA0011* -sgRNA-32 target sequence, R6K *ori* , Amp^r	This study
pB-iCRISPR-*cbuA0011* -sgRNA-33	pB-iCRISPR containing *cbuA0011-* sgRNA-33 target sequence, R6K *ori* , Amp^r	This study
pB-iCRISPR-*cbuA0012* -sgRNA-34	pB-iCRISPR containing *cbuA0012* -sgRNA-34 target sequence, R6K *ori* , Amp^r	This study
pB-iCRISPR-*cbuA0012* -sgRNA-35	pB-iCRISPR containing *cbuA0012* -sgRNA-35 target sequence, R6K *ori* , Amp^r	This study
pB-iCRISPR-*cbuA0012* -sgRNA-36	pB-iCRISPR containing *cbuA0012* -sgRNA-36 target sequence, R6K *ori* , Amp^r	This study
pB-iCRISPR-*cbuA0013* -sgRNA-37	pB-iCRISPR containing *cbuA0013* -sgRNA-37 target sequence, R6K *ori* , Amp^r	This study
pB-iCRISPR-*cbuA0013* -sgRNA-38	pB-iCRISPR containing *cbuA0013* -sgRNA-38 target sequence, R6K *ori* , Amp^r	This study
pB-iCRISPR-*cbuA0013* -sgRNA-39	pB-iCRISPR containing *cbuA0013* -sgRNA-39 target sequence, R6K *ori* , Amp^r	This study
pB-iCRISPR-*cbuA0013* a-sgRNA-40	pB-iCRISPR containing *cbuA0013a* -sgRNA-40 target sequence, R6K *ori* , Amp^r	This study
pB-iCRISPR-*cbuA0013a* -sgRNA-41	pB-iCRISPR containing *cbuA0013a* -sgRNA-41 target sequence, R6K *ori* , Amp^r	This study
pB-iCRISPR-*cbuA0013a* -sgRNA-42	pB-iCRISPR containing *cbuA0013a* -sgRNA-42 target sequence, R6K *ori* , Amp^r	This study
pB-iCRISPR-*cbuA0014* -sgRNA-43	pB-iCRISPR containing *cbuA0014* -sgRNA-43 target sequence, R6K *ori* , Amp^r	This study
pB-iCRISPR-*cbuA0014* -sgRNA-44	pB-iCRISPR containing *cbuA0014* -sgRNA-44 target sequence, R6K *ori* , Amp^r	This study
pB-iCRISPR-*cbuA0014* -sgRNA-45	pB-iCRISPR containing *cbuA0014* -sgRNA-45 target sequence, R6K *ori* , Amp^r	This study
pB-iCRISPR-*cbuA0015* -sgRNA-46	pB-iCRISPR containing *cbuA0015* -sgRNA-46 target sequence, R6K *ori* , Amp^r	This study
pB-iCRISPR-*cbuA0015* -sgRNA-47	pB-iCRISPR containing *cbuA0015* -sgRNA-47 target sequence, R6K *ori* , Amp^r	This study
pB-iCRISPR-*cbuA0015* -sgRNA-48	pB-iCRISPR containing *cbuA0015* -sgRNA-48 target sequence, R6K *ori* , Amp^r	This study
pB-iCRISPR-*cbuA0016* -sgRNA-49	pB-iCRISPR containing *cbuA0016* -sgRNA-49 target sequence, R6K *ori* , Amp^r	This study
pB-iCRISPR-*cbuA0016* -sgRNA-50	pB-iCRISPR containing *cbuA0016* -sgRNA-50 target sequence, R6K *ori* , Amp^r	This study
pB-iCRISPR-*cbuA0016* -sgRNA-51	pB-iCRISPR containing *cbuA0016* -sgRNA-51 target sequence, R6K *ori* , Amp^r	This study
pB-iCRISPR-*cbuA0017* -sgRNA-52	pB-iCRISPR containing *cbuA0017* -sgRNA-52 target sequence, R6K *ori* , Amp^r	This study
pB-iCRISPR-*cbuA0017* -sgRNA-53	pB-iCRISPR containing *cbuA0017* -sgRNA-53 target sequence, R6K *ori* , Amp^r	This study
pB-iCRISPR-*cbuA0017* -sgRNA-54	pB-iCRISPR containing *cbuA0017* -sgRNA-54 target sequence, R6K *ori* , Amp^r	This study
pB-iCRISPR-*cbuA0018* -sgRNA-55	pB-iCRISPR containing *cbuA0018* -sgRNA-55 target sequence, R6K *ori* , Amp^r	This study
pB-iCRISPR-*cbuA0018* -sgRNA-56	pB-iCRISPR containing *cbuA0018* -sgRNA-56 target sequence, R6K *ori* , Amp^r	This study
pB-iCRISPR-*cbuA0018* -sgRNA-57	pB-iCRISPR containing *cbuA0018* -sgRNA-57 target sequence, R6K *ori* , Amp^r	This study
pB-iCRISPR-*cbuA0020* -sgRNA-58	pB-iCRISPR containing *cbuA0020* -sgRNA-58 target sequence, R6K *ori* , Amp^r	This study
pB-iCRISPR-*cbuA0020* -sgRNA-59	pB-iCRISPR containing *cbuA0020* -sgRNA-59 target sequence, R6K *ori,* Amp^r	This study
pB-iCRISPR-*cbuA0020* -sgRNA-60	pB-iCRISPR containing *cbuA0020* -sgRNA-60 target sequence, R6K *ori* , Amp^r	This study
pB-iCRISPR-*cbuA0021* -sgRNA-61	pB-iCRISPR containing *cbuA0021* -sgRNA-61 target sequence, R6K *ori* , Amp^r	This study
pB-iCRISPR-*cbuA0021* -sgRNA-62	pB-iCRISPR containing *cbuA0021* -sgRNA-62 target sequence, R6K *ori* , Amp^r	This study
pB-iCRISPR-*cbuA0021* -sgRNA-63	pB-iCRISPR containing *cbuA0021* -sgRNA-63 target sequence, R6K *ori* , Amp^r	This study
pB-iCRISPR-*cbuA0022* -sgRNA-64	pB-iCRISPR containing *cbuA0022* -sgRNA-64 target sequence, R6K *ori* , Amp^r	This study
pB-iCRISPR-*cbuA0022* -sgRNA-65	pB-iCRISPR containing *cbuA0022* -sgRNA-65 target sequence, R6K *ori* , Amp^r	This study
pB-iCRISPR-*cbuA0022* -sgRNA-66	pB-iCRISPR containing *cbuA0022* -sgRNA-66 target sequence, R6K *ori,* Amp^r	This study
pB-iCRISPR-*cbuA0023* -sgRNA-67	pB-iCRISPR containing *cbuA0023* -sgRNA-67 target sequence, R6K *ori* , Amp^r	This study
pB-iCRISPR-*cbuA0023* -sgRNA-68	pB-iCRISPR containing *cbuA0023* -sgRNA-68 target sequence, R6K *ori* , Amp^r	This study
pB-iCRISPR-*cbuA0023* -sgRNA-69	pB-iCRISPR containing *cbuA0023* -sgRNA-69 target sequence, R6K *ori* , Amp^r	This study
pB-iCRISPR-*cbuA0023a* -sgRNA-70	pB-iCRISPR containing *cbuA0023a* -sgRNA-70 target sequence, R6K *ori* , Amp^r	This study
pB-iCRISPR-*cbuA0023a* -sgRNA-71	pB-iCRISPR containing *cbuA0023a* -sgRNA-71 target sequence, R6K *ori* , Amp^r	This study
pB-iCRISPR-*cbuA0024* -sgRNA-72	pB-iCRISPR containing *cbuA0024* -sgRNA-72 target sequence, R6K *ori* , Amp^r	This study
pB-iCRISPR-*cbuA0024* -sgRNA-73	pB-iCRISPR containing *cbuA0024* -sgRNA-73 target sequence, R6K *ori* , Amp^r	This study
pB-iCRISPR-*cbuA0024* -sgRNA-74	pB-iCRISPR containing *cbuA0024* -sgRNA-74 target sequence, R6K *ori* , Amp^r	This study
pB-iCRISPR-*cbuA0025* -sgRNA-75	pB-iCRISPR containing *cbuA0025* -sgRNA-75 target sequence, R6K *ori* , Amp^r	This study

pB-iCRISPR-*cbuA0025* -sgRNA-76	pB-iCRISPR containing *cbuA0025-* sgRNA-76 target sequence, R6K *ori* , Amp^r	This study
pB-iCRISPR-*cbuA0025* -sgRNA-77	pB-iCRISPR containing *cbuA0025* -sgRNA-77 target sequence, R6K *ori* , Amp^r	This study
pB-iCRISPR-*cbuA0026* -sgRNA-78	pB-iCRISPR containing *cbuA0026* -sgRNA-78 target sequence, R6K *ori* , Amp^r	This study
pB-iCRISPR-*cbuA0026* -sgRNA-79	pB-iCRISPR containing *cbuA0026* -sgRNA-79 target sequence, R6K *ori* , Amp^r	This study
pB-iCRISPR-*cbuA0026* -sgRNA-80	pB-iCRISPR containing *cbuA0026* -sgRNA-80 target sequence, R6K *ori* , Amp^r	This study
pB-iCRISPR-*cbuA0026a* -sgRNA-81	pB-iCRISPR containing *cbuA0026a* -sgRNA-81 target sequence, R6K *ori* , Amp^r	This study
pB-iCRISPR-*cbuA0026a* -sgRNA-82	pB-iCRISPR containing *cbuA0026a* -sgRNA-82 target sequence, R6K *ori* , Amp^r	This study
pB-iCRISPR-*cbuA0026a* -sgRNA-83	pB-iCRISPR containing *cbuA0026a* -sgRNA-83 target sequence, R6K *ori* , Amp^r	This study
pB-iCRISPR-*antitoxP* -sgRNA-84	pB-iCRISPR containing *cbuA0027* -sgRNA-84 target sequence, R6K *ori* , Amp^r	This study
pB-iCRISPR-*antitoxP* -sgRNA-85	pB-iCRISPR containing *cbuA0027* -sgRNA-85 target sequence, R6K *ori* , Amp^r	This study
pB-iCRISPR-*antitoxP* -sgRNA-86	pB-iCRISPR containing *cbuA0027* -sgRNA-86 target sequence, R6K *ori* , Amp^r	This study
pB-iCRISPR-*toxP* -sgRNA-87	pB-iCRISPR containing *cbuA0028* -sgRNA-87 target sequence, R6K *ori* , Amp^r	This study
pB-iCRISPR-*toxP* -sgRNA-88	pB-iCRISPR containing *cbuA0028* -sgRNA-88 target sequence, R6K *ori* , Amp^r	This study
pB-iCRISPR-*toxP* -sgRNA-89	pB-iCRISPR containing *cbuA0028* -sgRNA-89 target sequence, R6K *ori* , Amp^r	This study
pB-iCRISPR-*cbuA0029* -sgRNA-90	pB-iCRISPR containing *cbuA0029* -sgRNA-90 target sequence, R6K *ori* , Amp^r	This study
pB-iCRISPR-*cbuA0029* -sgRNA-91	pB-iCRISPR containing *cbuA0029* -sgRNA-91 target sequence, R6K *ori* , Amp^r	This study
pB-iCRISPR-*cbuA0029* -sgRNA-92	pB-iCRISPR containing *cbuA0029* -sgRNA-92 target sequence, R6K *ori* , Amp^r	This study
pB-iCRISPR-*cbuA0029a* -sgRNA-93	pB-iCRISPR containing *cbuA0029a* -sgRNA-93 target sequence, R6K *ori* , Amp^r	This study
pB-iCRISPR-*cbuA0029a* -sgRNA-94	pB-iCRISPR containing *cbuA0029a* -sgRNA-94 target sequence, R6K *ori* , Amp^r	This study
pB-iCRISPR-*cbuA0029a* -sgRNA-95	pB-iCRISPR containing *cbuA0029a* -sgRNA-95 target sequence, R6K *ori* , Amp^r	This study
pB-iCRISPR-*cbuA0031* -sgRNA-96	pB-iCRISPR containing *cbuA0031* -sgRNA-96 target sequence, R6K *ori,* Amp^r	This study
pB-iCRISPR-*cbuA0031* -sgRNA-97	pB-iCRISPR containing *cbuA0031* -sgRNA-97 target sequence, R6K *ori* , Amp^r	This study
pB-iCRISPR-*cbuA0031* -sgRNA-98	pB-iCRISPR containing *cbuA0031* -sgRNA-98 target sequence, R6K *ori* , Amp^r	This study
pB-iCRISPR-*cbuA0032* -sgRNA-99	pB-iCRISPR containing *cbuA0032* -sgRNA-99 target sequence, R6K *ori* , Amp^r	This study
pB-iCRISPR-*cbuA0032* -sgRNA-100	pB-iCRISPR containing *cbuA0032* -sgRNA-100 target sequence, R6K *ori* , Amp^r	This study
pB-iCRISPR-*cbuA0032* -sgRNA-101	pB-iCRISPR containing *cbuA0032* -sgRNA-101 target sequence, R6K *ori* , Amp^r	This study
pB-iCRISPR-*cbuA0033* -sgRNA-102	pB-iCRISPR containing *cbuA0033* -sgRNA-102 target sequence, R6K *ori* , Amp^r	This study
pB-iCRISPR-*cbuA0033* -sgRNA-103	pB-iCRISPR containing *cbuA0033* -sgRNA-103 target sequence, R6K *ori* , Amp^r	This study
pB-iCRISPR-*cbuA0033* -sgRNA-104	pB-iCRISPR containing *cbuA0033* -sgRNA-104 target sequence, R6K *ori* , Amp^r	This study
pB-iCRISPR-*cbuA0034* -sgRNA-105	pB-iCRISPR containing *cbuA0034* -sgRNA-105 target sequence, R6K *ori* , Amp^r	This study
pB-iCRISPR-*cbuA0034* -sgRNA-106	pB-iCRISPR containing *cbuA0034* -sgRNA-106 target sequence, R6K *ori* , Amp^r	This study
pB-iCRISPR-*cbuA0034* -sgRNA-107	pB-iCRISPR containing *cbuA0034* -sgRNA-107 target sequence, R6K *ori* , Amp^r	This study
pB-iCRISPR-*cbuA0034a* -sgRNA-108	pB-iCRISPR containing *cbuA0034a* -sgRNA-108 target sequence, R6K *ori* , Amp^r	This study
pB-iCRISPR-*cbuA0034a* -sgRNA-109	pB-iCRISPR containing *cbuA0034a* -sgRNA-109 target sequence, R6K *ori* , Amp^r	This study
pB-iCRISPR-*cbuA0034a* -sgRNA-110	pB-iCRISPR containing *cbuA0034a* -sgRNA-110 target sequence, R6K *ori* , Amp^r	This study
pB-iCRISPR-*cbuA0036* -sgRNA-111	pB-iCRISPR containing *cbuA0036* -sgRNA-111 target sequence, R6K *ori* , Amp^r	This study
pB-iCRISPR-*cbuA0036* -sgRNA-112	pB-iCRISPR containing *cbuA0036* -sgRNA-112 target sequence, R6K *ori* , Amp^r	This study
pB-iCRISPR-*cbuA0036* -sgRNA-113	pB-iCRISPR containing *cbuA0036* -sgRNA-113 target sequence, R6K *ori* , Amp^r	This study
pB-iCRISPR-*cbuA0036* -sgRNA-114	pB-iCRISPR containing *cbuA0036* -sgRNA-114 target sequence, R6K *ori* , Amp^r	This study
pB-iCRISPR-*cbuA0036* -sgRNA-115	pB-iCRISPR containing *cbuA0036* -sgRNA-115 target sequence, R6K *ori* , Amp^r	This study
pB-iCRISPR-*cbuA0037* -sgRNA-116	pB-iCRISPR containing *cbuA0037* -sgRNA-116 target sequence, R6K *ori* , Amp^r	This study
pB-iCRISPR-*cbuA0037* -sgRNA-117	pB-iCRISPR containing *cbuA0037* -sgRNA-117 target sequence, R6K *ori* , Amp^r	This study
pB-iCRISPR-*cbuA0037* -sgRNA-118	pB-iCRISPR containing *cbuA0037* -sgRNA-118 target sequence, R6K *ori* , Amp^r	This study
pB-iCRISPR-*cbuA0038* -sgRNA-119	pB-iCRISPR containing *cbuA0038* -sgRNA-119 target sequence, R6K ori, Amp^r	This study
pB-iCRISPR-*cbuA0038* -sgRNA-120	pB-iCRISPR containing *cbuA0038* -sgRNA-120 target sequence, R6K *ori* , Amp^r	This study
pB-iCRISPR-*cbuA0038* -sgRNA-121	pB-iCRISPR containing *cbuA0038* -sgRNA-121 target sequence, R6K *ori* , Amp^r	This study
pB-iCRISPR-*cbuA0039* -sgRNA-122	pB-iCRISPR containing *cbuA0039* -sgRNA-122 target sequence, R6K *ori,* Amp^r	This study
pB-iCRISPR-*cbuA0039* -sgRNA-123	pB-iCRISPR containing *cbuA0039* -sgRNA-123 target sequence, R6K *ori,* Amp^r	This study
pB-iCRISPR-*cbuA0039* -sgRNA-124	pB-iCRISPR containing *cbuA0039* -sgRNA-124 target sequence, R6K *ori* , Amp^r	This study
pB-iCRISPR-*cbuA0039* -sgRNA-125	pB-iCRISPR containing *cbuA0039* -sgRNA-125 target sequence, R6K *ori* , Amp^r	This study
pB-iCRISPR-*cbuA0039* -sgRNA-126	pB-iCRISPR containing *cbuA0039* -sgRNA-126 target sequence, R6K *ori* , Amp^r	This study

pB-iCRISPR-*cbuA0039a* -sgRNA127	pB-iCRISPR containing *cbuA0039a* -sgRNA127 target sequence, R6K *ori* , Amp^r	This study
pB-iCRISPR-*cbuA0039a* -sgRNA128	pB-iCRISPR containing *cbuA0039a* -sgRNA128 target sequence, R6K *ori* , Amp^r	This study
pB-iCRISPR-*cbuA0039a* -sgRNA129	pB-iCRISPR containing *cbuA0039a* -sgRNA129 target sequence, R6K *ori* , Amp^r	This study
CRISPRi complementation vectors		
pJB-*lysCA*	*lysCA* driven by *cbu1169* promoter replacing *cat* in pJB-CAT, Amp^r	Beare *et_al* 2018
pJB-*lysCA-tetRA*	*tetR* repressor and *tetA* promoter cloned into pJB-*lysCA* , Amp^r	This study
pJB-*lysCA-tetRA -icmD* -CO	pJB-*lysCA* containing codon optimized *cbu1624* , Amp^r	This study
pJB-*lysCA-tetRA-antitoxP* -CO	pJB-*lysCA* containing codon optimized *cbuA0027* , Amp^r	This study
antitoxP, toxP and toxPG C. burnetii expression vectors		
pMiniTn7T-*argGH*	Arginine-based nutritional selection vector, R6K *ori* , Amp^r	This study
pMiniTn7T-*argGH-lacIO*	pMiniTn7T-*argGH* containing lacI repressor and *lacO* operator from pJMP1356, R6K *ori* , Amp^r	This study
pMiniTn7T-*argGH-lacIO-antitoxP*	pMiniTn7T-*argGH-lacIO* containing *cbuA0027* cloned into the unique AvaI restriction site, R6K *ori* , Amp^r	This study
pMiniTn7T-CAT	R6K *ori* , Cm^r Amp^r	Beare *et_al* 2011
pMiniTn7T-*proBA*	*1169 ^{P} -proBA* cloned into pMiniTn7T-CAT, replacing *cat* , proline-based nutritional selection vector, R6K *ori* , Amp^r	This study
pMiniTn7T-*proBA -tetRA*	*tetR* repressor and *tetA* promoter cloned into pMiniTn7T-*proBA* , R6K *ori* , Amp^r	This study
pMiniTn7T-*proBA-tetRA-toxP*	*cbuA0028* from NMII cloned into pMiniTn7T-*proBA-tetRA* , R6K *ori* , Amp^r	This study
pMiniTn7T-*proBA-tetRA-toxP* G	*cbuA0028* from the G Q212 strain cloned into pMiniTn7T-*proBA-tetRA* , R6K *ori* , Amp^r	This study
antitoxP, toxP and cbu0665 cell-free expression vectors		
pET28a(+)	*E. coli* expression vector with C-terminal V5 and 6xHis tag, pMB1 *ori* , Km^r	Millipore Sigma
pET28a(+)-*toxP*	*cbuA0028* -V5 cloned into pET28a(+), pMB1 *ori* , Km^r	This study
pDEST15	N-terminal GST tagged vector, Cm^r, Amp^r	Invitrogen
pDEST15-*antitoxP*	*cbuA0027* cloned into pDEST15, Amp^r	This study
pEXP1-DEST	N-terminal XpressT and 6xHis tagged vector, Cm^r, Amp^r	Invitrogen
pEXP1-*cbu0665*	*cbu0665* cloned into pEXP1-DEST, Amp^r	This study
toxP and antitoxP promoter analysis vectors		
pMiniTn7T-*lysCA*	Lysine-based nutritional selection vector, R6K *ori* , Amp^r	This study
pmScarlet-i_C1	Mammalian expression vector of *mScarlet-i* , Km^r	Bindels *et_al* 2017
pMiniTn7T-*lysCA* -NoP-*mScarlet-i*	Promoterless *mScarlet-i* cloned into pMiniTn7T-*lysCA* , R6K *ori* , Amp^r	This study
pMiniTn7T-*lysCA* -P*antitoxP* -Frag-1-*mScarlet-i*	Frag-1 *cbuA0027* promoter fused to *mScarlet-i* and cloned into pMiniTn7T-*lysCA* , R6K *ori* , Amp^r	This study
pMiniTn7T-*lysCA* -P*antitoxP* -Frag-2-*mScarlet-i*	Frag-2 *cbuA0027* promoter fused to *mScarlet-i* and cloned into pMiniTn7T-*lysCA* , R6K *ori* , Amp^r	This study
pMiniTn7T-*lysCA* -P*toxP* -*mScarlet-i*	*cbuA0028* promoter fused to *mScarlet-i* and cloned into pMiniTn7T-*lysCA* , R6K *ori* , Amp^r	This study

BEARE, P. A., GILK, S. D., LARSON, C. L., HILL, J., STEAD, C. M., OMSLAND, A., COCKRELL, D. C., HOWE, D., VOTH, D. E. & HEINZEN, R. A. 2011. Dot/Icm type IVB secretion system requirements for *Coxiella burnetii* growth in human macrophages. MBio, 2, e00175-11.

BEARE, P. A., JEFFREY, B. M., LONG, C. M., MARTENS, C. M. & HEINZEN, R. A. 2018. Genetic mechanisms of *Coxiella burnetii* lipopolysaccharide phase variation. PLoS Pathog, 14, e1006922.

BEARE, P. A., LARSON, C. L., GILK, S. D. & HEINZEN, R. A. 2012. Two systems for targeted gene deletion in *Coxiella burnetii* . Appl Environ Microbiol, 78, 4580-9.

BINDELS, D. S., HAARBOSCH, L., VAN WEEREN, L., POSTMA, M., WIESE, K. E., MASTOP, M., AUMONIER, S., GOTTHARD, G., ROYANT, A., HINK, M. A. & GADELLA, T. W., JR. 2017. mScarlet: a bright monomeric red fluorescent protein for cellular imaging. Nat Methods, 14, 53-56.

OMSLAND, A., BEARE, P. A., HILL, J., COCKRELL, D. C., HOWE, D., HANSEN, B., SAMUEL, J. E. & HEINZEN, R. A. 2011. Isolation from animal tissue and genetic transformation of *Coxiella burnetii* are facilitated by an improved axenic growth medium. Appl Environ Microbiol, 77, 3720-5.

PETERS, J. M., KOO, B. M., PATINO, R., HEUSSLER, G. E., HEARNE, C. C., QU, J., INCLAN, Y. F., HAWKINS, J. S., LU, C. H. S., SILVIS, M. R., HARDEN, M. M., OSADNIK, H., PETERS, J. E., ENGEL, J. N., DUTTON, R. J., GROSSMAN, A. D., GROSS, C. A. & ROSENBERG, O. S. 2019. Enabling genetic analysis of diverse bacteria with Mobile-CRISPRi. Nat Microbiol, 4, 244-250.

Primer name	Sequence (5' - 3')
Coxiella shuttle vector construction	
P1169-CAT-SacB-forQpH1plasmid-F	CTGATGCGGTATTTTGGACAATGTTTTTCTACCAAAGC
P1169-CAT-SacB-forQpH1plasmid-R	TTATTTGTTAACTGTTAATTGTCCTTG
P1169-TyrB-forQpH1plasmid-F	ACAGTTAACAAATAAATGGCTTCGTTTCGCAG
P1169-TyrB-forQpH1plasmid-R	TTACATCACCGCAGCAAAC
pBR322ori-forQpH1plasmid-F	GCTGCGGTGATGTAACCTGCAGGTTAACGTGAGTTTTCGTTCCA
pBR322ori-forQpH1plasmid-R	AAAATACCGCATCAGGCG
QpH1ori-forpBR322-F	TGCGGTGATGTAACCTGCAGGTTCTATAGAAGGCTTTGCTAAATC
QpH1ori-forpBR322-R	AAACTCACGTTAACCTATGGATAAAATCAAATCAATTC
PCR analysis of ΔQpH1	
CBUA0023-F	TTACTCAATGGAATTCAAGATATGGTGAAGGAAATTTAAC
CBUA0023-R	GCTTCTCGAGGAATTCAGATATTGAGGAGGATTTTAGATTC
CAT-detection-F	ATGGAGAAAAAAATCACTGGATATACCACC
CAT-detection-R	TTACGCCCCGCCCTGCCACTCATCGC
CRISPRi primers	
proBA-forpJMP-F	CTGTTGAACTCTCGAGATGGCTTCGTTTCGCAGC
proBA-BsaIfix-F	TTGTCTCAAAAGAATGTGATTATC
proBA-BsaIfix-R	ATTCTTTTGAGACAACGTCTCCACAAGTTCTGG
proBA-forpJMP-R1	CTAAACAAAAAAACCCTGTCACTTTTTACAGTGACAGGGTTTTTAATATATCAATCTCTAATTTGTCCTG
proBA-forpJMP-R2	GAAGCTGATGCTCGAGCTAAACAAAAAAACCCTGTCACTTTTTAC
CBU1624-sgRNA-3-F	tagtATAACACCAAAAGCGGACCT
CBU1624-sgRNA-3-R	aaacAGGTCCGCTTTTGGTGTTAT
CBU1624-sgRNA-5-F	tagtACTCCAGATAACACCAAAAG
CBU1624-sgRNA-5-R	aaacCTTTTGGTGTTATCTGGAGT
CBU1267a-sgRNA-9-F	tagtTTGGACATTTTGTCTTTCCA
CBU1267a-sgRNA-9-R	aaacTGGAAAGACAAAATGTCCAA
CBUA0001-sgRNA-1-F	tagtCCGCTGATCGAGCTGTTTTA
CBUA0001-sgRNA-1-R	aaacTAAAACAGCTCGATCAGCGG
CBUA0001-sgRNA-2-F	tagtATGGATGCCCTGTTCTTTGA
CBUA0001-sgRNA-2-R	aaacTCAAAGAACAGGGCATCCAT
CBUA0001-sgRNA-3-F	tagtAATGGATGCCCTGTTCTTTG
CBUA0001-sgRNA-3-R	aaacCAAAGAACAGGGCATCCATT
CBUA0003-sgRNA-4-F	tagtCACTTTTTTGACAAAATCAA
CBUA0003-sgRNA-4-R	aaacTTGATTTTGTCAAAAAAGTG
CBUA0003-sgRNA-5-F	tagtGCACTTTTTTGACAAAATCA
CBUA0003-sgRNA-5-R	aaacTGATTTTGTCAAAAAAGTGC
CBUA0003-sgRNA-6-F	tagtACGTTTTCCCGAACATTCGG
CBUA0003-sgRNA-6-R	aaacCCGAATGTTCGGGAAAACGT
CBUA0003a-sgRNA-7-F	tagtTTCATTTGCTGCCAATGCGT
CBUA0003a-sgRNA-7-R	aaacACGCATTGGCAGCAAATGAA
CBUA0003a-sgRNA-8-F	tagtCAGCCGCCTCATACAGTTTC
CBUA0003a-sgRNA-8-R	aaacGAAACTGTATGAGGCGGCTG
CBUA0003a-sgRNA-9-F	tagtAGAGTCCTCAATTGACAGGC
CBUA0003a-sgRNA-9-R	aaacGCCTGTCAATTGAGGACTCT
CBUA0005-sgRNA-10-F	tagtAACGTGTTCTTAGTAGCTAT
CBUA0005-sgRNA-10-R	aaacATAGCTACTAAGAACACGTT
CBUA0005-sgRNA-11-F	tagtTCGCGTGTTTTCACTGGTGG
CBUA0005-sgRNA-11-R	aaacCCACCAGTGAAAACACGCGA
CBUA0005-sgRNA-12-F	tagtCAATCGCGTGTTTTCACTGG
CBUA0005-sgRNA-12-R	aaacCCAGTGAAAACACGCGATTG
CBUA0006-sgRNA-13-F	tagtAAACCAGCTGTTTGGCTAGA
CBUA0006-sgRNA-13-R	aaacTCTAGCCAAACAGCTGGTTT
CBUA0006-sgRNA-14-F	tagtTTGCAAACAAACCAGCTGTT
CBUA0006-sgRNA-14-R	aaacAACAGCTGGTTTGTTTGCAA

CBUA0006-sgRNA-15-F	tagtATCATGATATCCTTTAAGGA
CBUA0006-sgRNA-15-R	aaacTCCTTAAAGGATATCATGAT
CBUA0007-sgRNA-16-F	tagtAATAGAAGTTAGAAAAGTAT
CBUA0007-sgRNA-16-R	aaacATACTTTTCTAACTTCTATT
CBUA0007-sgRNA-17-F	tagtCAATAGAAGTTAGAAAAGTA
CBUA0007-sgRNA-17-R	aaacTACTTTTCTAACTTCTATTG
CBUA0007-sgRNA-18-F	tagtGAATGATTCAGCACGCTGTC
CBUA0007-sgRNA-18-R	aaacGACAGCGTGCTGAATCATTC
CBUA0008-sgRNA-19-F	tagtGGTCCGCTATAAAAGGGGGG
CBUA0008-sgRNA-19-R	aaacCCCCCCTTTTATAGCGGACC
CBUA0008-sgRNA-20-F	tagtTGTGGTCCGCTATAAAAGGG
CBUA0008-sgRNA-20-R	aaacCCCTTTTATAGCGGACCACA
CBUA0008-sgRNA-21-F	tagtATGTGGTCCGCTATAAAAGG
CBUA0008-sgRNA-21-R	aaacCCTTTTATAGCGGACCACAT
CBUA0008a-sgRNA-22-F	tagtGCTTGGTTGGTAGGATGTCC
CBUA0008a-sgRNA-22-R	aaacGGACATCCTACCAACCAAGC
CBUA0008a-sgRNA-23-F	tagtTAAATCCTTGCTTGGTTGGT
CBUA0008a-sgRNA-23-R	aaacACCAACCAAGCAAGGATTTA
CBUA0008a-sgRNA-24-F	tagtAGCGTAAATCCTTGCTTGGT
CBUA0008a-sgRNA-24-R	aaacACCAAGCAAGGATTTACGCT
CBUA0008b-sgRNA-25-F	tagtAAATTTGTCGCTTGGGTATA
CBUA0008b-sgRNA-25-R	aaacTATACCCAAGCGACAAATTT
CBUA0008b-sgRNA-26-F	tagtACTGATAAAATTTGTCGCTT
CBUA0008b-sgRNA-26-R	aaacAAGCGACAAATTTTATCAGT
CBUA0008b-sgRNA-27-F	tagtCACTGATAAAATTTGTCGCT
CBUA0008b-sgRNA-27-R	aaacAGCGACAAATTTTATCAGTG
CBUA0010-sgRNA-28-F	tagtAGTGGTACTGTTTTCATCAA
CBUA0010-sgRNA-28-R	aaacTTGATGAAAACAGTACCACT
CBUA0010-sgRNA-29-F	tagtATAGAGTCAAATAGTGCTAG
CBUA0010-sgRNA-29-R	aaacCTAGCACTATTTGACTCTAT
CBUA0010-sgRNA-30-F	tagtAGTCCCTTGGCTACCTTTGT
CBUA0010-sgRNA-30-R	aaacACAAAGGTAGCCAAGGGACT
CBUA0011-sgRNA-31-F	tagtAGCGGTGCACCTGAGTCCAA
CBUA0011-sgRNA-31-R	aaacTTGGACTCAGGTGCACCGCT
CBUA0011-sgRNA-32-F	tagtTTTTCTTGGGCGACTTTAAG
CBUA0011-sgRNA-32-R	aaacCTTAAAGTCGCCCAAGAAAA
CBUA0011-sgRNA-33-F	tagtACTATGTCCAGCATTTTCTT
CBUA0011-sgRNA-33-R	aaacAAGAAAATGCTGGACATAGT
CBUA0012-sgRNA-34-F	tagtGCCCCACAGAAGTCTTGCCG
CBUA0012-sgRNA-34-R	aaacCGGCAAGACTTCTGTGGGGC
CBUA0012-sgRNA-35-F	tagtAAATAACTTCTCGGCATAAT
CBUA0012-sgRNA-35-R	aaacATTATGCCGAGAAGTTATTT
CBUA0012-sgRNA-36-F	tagtGCATTCCCAAAATAACTTCT
CBUA0012-sgRNA-36-R	aaacAGAAGTTATTTTGGGAATGC
CBUA0013-sgRNA-37-F	tagtTTCGGTAGTGTAAAAAAATA
CBUA0013-sgRNA-37-R	aaacTATTTTTTTACACTACCGAA
CBUA0013-sgRNA-38-F	tagtTTTTCTTTTATGTCAATTTT
CBUA0013-sgRNA-38-R	aaacAAAATTGACATAAAAGAAAA
CBUA0013-sgRNA-39-F	tagtTTAAAACTTTTTCTGGAGTT
CBUA0013-sgRNA-39-R	aaacAACTCCAGAAAAAGTTTTAA
CBUA0013a-sgRNA-40-F	tagtAGGCTGAATTCTACACGTCA
CBUA0013a-sgRNA-40-R	aaacTGACGTGTAGAATTCAGCCT
CBUA0013a-sgRNA-41-F	tagtCAGGCTGAATTCTACACGTC
CBUA0013a-sgRNA-41-R	aaacGACGTGTAGAATTCAGCCTG
CBUA0013a-sgRNA-42-F	tagtTGCTTGCGATCGAGATCCTC
CBUA0013a-sgRNA-42-R	aaacGAGGATCTCGATCGCAAGCA
CBUA0014-sgRNA-43-F	tagtGGACACATCTTCATTCGTTT
CBUA0014-sgRNA-43-R	aaacAAACGAATGAAGATGTGTCC
CBUA0014-sgRNA-44-F	tagtTAAATGATCCCTTTCAGAAG
CBUA0014-sgRNA-44-R	aaacCTTCTGAAAGGGATCATTTA

CBUA0014-sgRNA-45-F	tagtATAAATGATCCCTTTCAGAA
CBUA0014-sgRNA-45-R	aaacTTCTGAAAGGGATCATTTAT
CBUA0015-sgRNA-46-F	tagtACCGTGCAATTTTTAAATTC
CBUA0015-sgRNA-46-R	aaacGAATTTAAAAATTGCACGGT
CBUA0015-sgRNA-47-F	tagtAATGATTAAACCGGCCCGGT
CBUA0015-sgRNA-47-R	aaacACCGGGCCGGTTTAATCATT
CBUA0015-sgRNA-48-F	tagtCAATGATTAAACCGGCCCGG
CBUA0015-sgRNA-48-R	aaacCCGGGCCGGTTTAATCATTG
CBUA0016-sgRNA-49-F	tagtCTTCTTTTGAGTGAATTTCT
CBUA0016-sgRNA-49-R	aaacAGAAATTCACTCAAAAGAAG
CBUA0016-sgRNA-50-F	tagtTTGAATCTTGTGGTAAGCTA
CBUA0016-sgRNA-50-R	aaacTAGCTTACCACAAGATTCAA
CBUA0016-sgRNA-51-F	tagtTTTGAATCTTGTGGTAAGCT
CBUA0016-sgRNA-51-R	aaacAGCTTACCACAAGATTCAAA
CBUA0017-sgRNA-52-F	tagtCGCAGAACGCATAGAGGAAA
CBUA0017-sgRNA-52-R	aaacTTTCCTCTATGCGTTCTGCG
CBUA0017-sgRNA-53-F	tagtGCGCAGAACGCATAGAGGAA
CBUA0017-sgRNA-53-R	aaacTTCCTCTATGCGTTCTGCGC
CBUA0017-sgRNA-54-F	tagtACAGCGCGCAGAACGCATAG
CBUA0017-sgRNA-54-R	aaacCTATGCGTTCTGCGCGCTGT
CBUA0018-sgRNA-55-F	tagtAACCATCGTATCCTTTTTTA
CBUA0018-sgRNA-55-R	aaacTAAAAAAGGATACGATGGTT
CBUA0018-sgRNA-56-F	tagtGATCTTCATAGGGGCGAGGG
CBUA0018-sgRNA-56-R	aaacCCCTCGCCCCTATGAAGATC
CBUA0018-sgRNA-57-F	tagtATTGATCTTCATAGGGGCGA
CBUA0018-sgRNA-57-R	aaacTCGCCCCTATGAAGATCAAT
CBUA0020-sgRNA-58-F	tagtGCTCAATCAATACTGCATTT
CBUA0020-sgRNA-58-R	aaacAAATGCAGTATTGATTGAGC
CBUA0020-sgRNA-59-F	tagtATGGGGGGTAATTCTTTTGG
CBUA0020-sgRNA-59-R	aaacCCAAAAGAATTACCCCCCAT
CBUA0020-sgRNA-60-F	tagtAAAATGGGGGGTAATTCTTT
CBUA0020-sgRNA-60-R	aaacAAAGAATTACCCCCCATTTT
CBUA0021-sgRNA-61-F	tagtACAGTAGGATTATTAGGAGG
CBUA0021-sgRNA-61-R	aaacCCTCCTAATAATCCTACTGT
CBUA0021-sgRNA-62-F	tagtCTGACAGTAGGATTATTAGG
CBUA0021-sgRNA-62-R	aaacCCTAATAATCCTACTGTCAG
CBUA0021-sgRNA-63-F	tagtCTACTGACAGTAGGATTATT
CBUA0021-sgRNA-63-R	aaacAATAATCCTACTGTCAGTAG
CBUA0022-sgRNA-64-F	tagtAGGCGCATTGTAGGCAAAAA
CBUA0022-sgRNA-64-R	aaacTTTTTGCCTACAATGCGCCT
CBUA0022-sgRNA-65-F	tagtTAGGCGCATTGTAGGCAAAA
CBUA0022-sgRNA-65-R	aaacTTTTGCCTACAATGCGCCTA
CBUA0022-sgRNA-66-F	tagtTAAGTCCATAGGCGCATTGT
CBUA0022-sgRNA-66-R	aaacACAATGCGCCTATGGACTTA
CBUA0023-sgRNA-67-F	tagtATGTGCTTTTTCAACACTAT
CBUA0023-sgRNA-67-R	aaacATAGTGTTGAAAAAGCACAT
CBUA0023-sgRNA-68-F	tagtAGGGAAGGATTGGTTTTCCG
CBUA0023-sgRNA-68-R	aaacCGGAAAACCAATCCTTCCCT
CBUA0023-sgRNA-69-F	tagtTAGGGAAGGATTGGTTTTCC
CBUA0023-sgRNA-69-R	aaacGGAAAACCAATCCTTCCCTA
CBUA0023a-sgRNA-70-F	tagtTTATACTGAGAACCAAGGTT
CBUA0023a-sgRNA-70-R	aaacAACCTTGGTTCTCAGTATAA
CBUA0023a-sgRNA-71-F	tagtGCTTTTTATACTGAGAACCA
CBUA0023a-sgRNA-71-R	aaacTGGTTCTCAGTATAAAAAGC
CBUA0024-sgRNA-72-F	tagtCGATAGGGCCAATGGGGATT
CBUA0024-sgRNA-72-R	aaacAATCCCCATTGGCCCTATCG
CBUA0024-sgRNA-73-F	tagtCGGGATCGATAGGGCCAATG
CBUA0024-sgRNA-73-R	aaacCATTGGCCCTATCGATCCCG
CBUA0024-sgRNA-74-F	tagtTCGGGATCGATAGGGCCAAT
CBUA0024-sgRNA-74-R	aaacATTGGCCCTATCGATCCCGA

CBUA0025-sgRNA-75-F	tagtAAGAATTTCACCCCATCATT
CBUA0025-sgRNA-75-R	aaacAATGATGGGGTGAAATTCTT
CBUA0025-sgRNA-76-F	tagtTTTTTGGTTTTTTACCACCT
CBUA0025-sgRNA-76-R	aaacAGGTGGTAAAAAACCAAAAA
CBUA0025-sgRNA-77-F	tagtTTTTTTGGTTTTTTACCACC
CBUA0025-sgRNA-77-R	aaacGGTGGTAAAAAACCAAAAAA
CBUA0026-sgRNA-78-F	tagtGGATTTCAGAGGTTAAAACT
CBUA0026-sgRNA-78-R	aaacAGTTTTAACCTCTGAAATCC
CBUA0026-sgRNA-79-F	tagtTAGGCATACAGGGATTTCAG
CBUA0026-sgRNA-79-R	aaacCTGAAATCCCTGTATGCCTA
CBUA0026-sgRNA-80-F	tagtGTTTAAGTTTTAGGCATACA
CBUA0026-sgRNA-80-R	aaacTGTATGCCTAAAACTTAAAC
CBUA0026a-sgRNA-81-F	tagtCATATACACACATCAACACT
CBUA0026a-sgRNA-81-R	aaacAGTGTTGATGTGTGTATATG
CBUA0026a-sgRNA-82-F	tagtTTTTGCCGACTCAGACTCAA
CBUA0026a-sgRNA-82-R	aaacTTGAGTCTGAGTCGGCAAAA
CBUA0026a-sgRNA-83-F	tagtCAGATTGTTTAATTTTTTCT
CBUA0026a-sgRNA-83-R	aaacAGAAAAAATTAAACAATCTG
CBUA0027-sgRNA-84-F	tagtACGGCCTCAATGGGCGATAA
CBUA0027-sgRNA-84-R	aaacTTATCGCCCATTGAGGCCGT
CBUA0027-sgRNA-85-F	tagtGATTCATGAACGGCCTCAAT
CBUA0027-sgRNA-85-R	aaacATTGAGGCCGTTCATGAATC
CBUA0027-sgRNA-86-F	tagtAGATTCATGAACGGCCTCAA
CBUA0027-sgRNA-86-R	aaacTTGAGGCCGTTCATGAATCT
CBUA0028-sgRNA-87-F	tagtTTTCTGATTTTGCCCATCGA
CBUA0028-sgRNA-87-R	aaacTCGATGGGCAAAATCAGAAA
CBUA0028-sgRNA-88-F	tagtGGCCGTTTTCAGTTGGCTAT
CBUA0028-sgRNA-88-R	aaacATAGCCAACTGAAAACGGCC
CBUA0028-sgRNA-89-F	tagtCACTAATGGCCGTTTTCAGT
CBUA0028-sgRNA-89-R	aaacACTGAAAACGGCCATTAGTG
CBUA0029-sgRNA-90-F	tagtGACCATTTAAGAGTGTGGGC
CBUA0029-sgRNA-90-R	aaacGCCCACACTCTTAAATGGTC
CBUA0029-sgRNA-91-F	tagtTTTTGACCATTTAAGAGTGT
CBUA0029-sgRNA-91-R	aaacACACTCTTAAATGGTCAAAA
CBUA0029-sgRNA-92-F	tagtTTTTTGACCATTTAAGAGTG
CBUA0029-sgRNA-92-R	aaacCACTCTTAAATGGTCAAAAA
CBUA0029a-sgRNA-93-F	tagtTGCTTCACGTGAGGCAATCA
CBUA0029a-sgRNA-93-R	aaacTGATTGCCTCACGTGAAGCA
CBUA0029a-sgRNA-94-F	tagtGGTAAATGTTGCTTCACGTG
CBUA0029a-sgRNA-94-R	aaacCACGTGAAGCAACATTTACC
CBUA0029a-sgRNA-95-F	tagtTTTTTCAACGACGGATTGAT
CBUA0029a-sgRNA-95-R	aaacATCAATCCGTCGTTGAAAAA
CBUA0031-sgRNA-96-F	tagtTAAAAGAAAATGGAAATAAC
CBUA0031-sgRNA-96-R	aaacGTTATTTCCATTTTCTTTTA
CBUA0031-sgRNA-97-F	tagtATGAGTAATATAAAAGAAAA
CBUA0031-sgRNA-97-R	aaacTTTTCTTTTATATTACTCAT
CBUA0031-sgRNA-98-F	tagtTGGTGGAGAAGGGAGGCGTG
CBUA0031-sgRNA-98-R	aaacCACGCCTCCCTTCTCCACCA
CBUA0032-sgRNA-99-F	tagtTAAGTGCAAATCACTGACTT
CBUA0032-sgRNA-99-R	aaacAAGTCAGTGATTTGCACTTA
CBUA0032-sgRNA-100-F	tagtTTAAGTGCAAATCACTGACT
CBUA0032-sgRNA-100-R	aaacAGTCAGTGATTTGCACTTAA
CBUA0032-sgRNA-101-F	tagtATTGCTGTATCTGCCTCTAG
CBUA0032-sgRNA-101-R	aaacCTAGAGGCAGATACAGCAAT
CBUA0033-sgRNA-102-F	tagtTTAAGATAGAGTAAATCTTT
CBUA0033-sgRNA-102-R	aaacAAAGATTTACTCTATCTTAA
CBUA0033-sgRNA-103-F	tagtAACTCGAACCCGGTATCTTT
CBUA0033-sgRNA-103-R	aaacAAAGATACCGGGTTCGAGTT
CBUA0033-sgRNA-104-F	tagtTCCGCGCATAAACTCGAACC
CBUA0033-sgRNA-104-R	aaacGGTTCGAGTTTATGCGCGGA

CBUA0034-sgRNA-105-F	tagtGCTACGATTTTTGTATTTGA
CBUA0034-sgRNA-105-R	aaacTCAAATACAAAAATCGTAGC
CBUA0034-sgRNA-106-F	tagtTGCTACGATTTTTGTATTTG
CBUA0034-sgRNA-106-R	aaacCAAATACAAAAATCGTAGCA
CBUA0034-sgRNA-107-F	tagtTATCATCAGAGGATTAGTGT
CBUA0034-sgRNA-107-R	aaacACACTAATCCTCTGATGATA
CBUA0034a-sgRNA-108-F	tagtAATTTGCCCACTTTAACCTT
CBUA0034a-sgRNA-108-R	aaacAAGGTTAAAGTGGGCAAATT
CBUA0034a-sgRNA-109-F	tagtAAGCATTTGGAATTGCTCTA
CBUA0034a-sgRNA-109-R	aaacTAGAGCAATTCCAAATGCTT
CBUA0034a-sgRNA-110-F	tagtCTTCAAAAGCGTAAAGCATT
CBUA0034a-sgRNA-110-R	aaacAATGCTTTACGCTTTTGAAG
CBUA0036-sgRNA-111-F	tagtAACGAAATCGTTTGTTTTAG
CBUA0036-sgRNA-111-R	aaacCTAAAACAAACGATTTCGTT
CBUA0036-sgRNA-112-F	tagtACCCGTCTGTAGTAAATCAA
CBUA0036-sgRNA-112-R	aaacTTGATTTACTACAGACGGGT
CBUA0036-sgRNA-113-F	tagtATTCTTTTATCCAACCCTTT
CBUA0036-sgRNA-113-R	aaacCGAAAGGGTTGGATAAAAGA
CBUA0036-sgRNA-114-F	tagtTTCTTTTATCCAACCCTTTC
CBUA0036-sgRNA-114-R	aaacGAAAGGGTTGGATAAAAGAA
CBUA0036-sgRNA-115-F	tagtTCTTTTATCCAACCCTTTCG
CBUA0036-sgRNA-115-R	aaacATTCTTTTATCCAACCCTTT
CBUA0037-sgRNA-116-F	tagtGGGGGTTTCTGTACCGTAGG
CBUA0037-sgRNA-116-R	aaacCCTACGGTACAGAAACCCCC
CBUA0037-sgRNA-117-F	tagtCGGGGGTTTCTGTACCGTAG
CBUA0037-sgRNA-117-R	aaacCTACGGTACAGAAACCCCCG
CBUA0037-sgRNA-118-F	tagtTCGGGGGTTTCTGTACCGTA
CBUA0037-sgRNA-118-R	aaacTACGGTACAGAAACCCCCGA
CBUA0038-sgRNA-119-F	tagtTTTTTCATTAACATACCTAA
CBUA0038-sgRNA-119-R	aaacTTAGGTATGTTAATGAAAAA
CBUA0038-sgRNA-120-F	tagtAATTTTCAATTTTTTTAATC
CBUA0038-sgRNA-120-R	aaacGATTAAAAAAATTGAAAATT
CBUA0038-sgRNA-121-F	tagtTGCTGCTTTATTTAATACGA
CBUA0038-sgRNA-121-R	aaacTCGTATTAAATAAAGCAGCA
CBUA0039-sgRNA-122-F	tagtCAATCCGGGAGCTCCGGATT
CBUA0039-sgRNA-122-R	aaacAATCCGGAGCTCCCGGATTG
CBUA0039-sgRNA-123-F	tagtCCAATCCGGGAGCTCCGGAT
CBUA0039-sgRNA-123-R	aaacATCCGGAGCTCCCGGATTGG
CBUA0039-sgRNA-124-F	tagtAAGCTCCAATCCGGGAGCTC
CBUA0039-sgRNA-124-R	aaacGAGCTCCCGGATTGGAGCTT
CBUA0039-sgRNA-125-F	tagtTTTGGCTCAAGCTCCAATCC
CBUA0039-sgRNA-125-R	aaacGGATTGGAGCTTGAGCCAAA
CBUA0039-sgRNA-126-F	tagtATTTGGCTCAAGCTCCAATC
CBUA0039-sgRNA-126-R	aaacGATTGGAGCTTGAGCCAAAT
CBUA0039a-sgRNA-127-F	tagtGCATTGGGTTTGTGGCGCGT
CBUA0039a-sgRNA-127-R	aaacACGCGCCACAAACCCAATGC
CBUA0039a-sgRNA-128-F	tagtTTTTTTGGGCATTGGGTTTG
CBUA0039a-sgRNA-128-R	aaacCAAACCCAATGCCCAAAAAA
CBUA0039a-sgRNA-129-F	tagtCGGTATTTTTTTTGGGCATT
CBUA0039a-sgRNA-129-R	aaacAATGCCCAAAAAAAATACCG
CRISPRi complementation vector primers	
TetRA-forpJB-F	ACTGACGCGTGAATTCTTAAGACCCACTTTCACATTTAA
TetRA-forpJB-R	TCGTATGGGTACATCTGCAGCTTTTCTCTATCACTGATAGGG
CBU1624-CO-F1	AATACTCTTGGGCCCATTGCTCGTTCTCAGCGGCGTCATCGGCGTATTATTTGCTGGAAC
CBU1624-CO-F2	GTGATAGAGAAAAGCTGCAGATGCAGACTAAAACAATGCGCTTATTTCGAATACTCTTGGGCCC ATTGC
CBU1624-CO-R	GCATGCCTCAGTCGACTTATGACGAACCAATAATACTGTGG

CBUA0027-CO-F1	GTGATAGAGAAAAGCTGCAGGTGATGAAAGATAAAAAAAAGAAAAGTAAAACTCGTTTTAAAC CTCTGAGCCCAATCG
CBUA0027-CO-F2	TTAAACCTCTGAGCCCAATCGAAGCTGTGCACGAGAGCGCAAAAGGATTATATGATGCCG
CBUA0027-CO-R	CCTTCAAGCCATCGCTTAGGTCGACTGAGGCATGC
qRT-PCR probes and primers	
groEL-F	ATGGCTTACCCGCTTTCTC
groEL-R	AACGTCTTTATGGTTCAAGACTTT
groEL-probe	CalFluorGold-TGTTCAAGCAGCCGTTGTCGC-BHQ1
CBUA0006-F	CGCCAGTGCTCAACACATTC
CBUA0006-R	CACTGATTGATGCCTTACAAACCT
CBUA0006-probe	FAM-TCAAAGCTCCAACGCTCCACATTAGTTACG-BHQ1
CBUA0013-F	TCATTTTGGTGGTATTAACGGGTAT
CBUA0013-R	CTCCACTTCACCCTTGTTTTTTACTC
CBUA0013-probe	FAM-ATCCTCCAGAATGGAAAATCGCCGC-BHQ1
CBUA0014-F	CCTTCTGAAAGGGATCATTTATCC
CBUA0014-R	TGCTCAAATTCCGCACCTACTT
CBUA0014-probe	FAM-ATCTTTCTGCGCGGGATTCGTATTCTCT-BHQ1
CBUA0015-F	CCGGGCCGGTTTAATCAT
CBUA0015-R	GCTTTTACCACCCCGAAATTC
CBUA0015-probe	FAM-CGGAATTGACTGACTGCTCTCCCAGTG-BHQ1
CBUA0016-F	GGATAAAAACCAAACTCTCCTCCTT
CBUA0016-R	CCTCATTAGTGGATTCATCTCGATCT
CBUA0016-probe	FAM-TCGACCCTTGGAGCCAGGACAATAAA-BHQ1
CBUA0021-F	CTTGCCGGTTTTGATTACTCCTT
CBUA0021-R	GCTCGATCACGAAGAGAATATTTCT
CBUA0021-probe	FAM-TTTCTAAACACGTTCAATGCAGCCAGCA-BHQ1
CBUA0023-F	TTTTTATGGTTGCGAGGTTGTAT
CBUA0023-R	GGTTGAAGTCAGAGCCGGTTAT
CBUA0023-probe	FAM-CTTCTCCAACCGAGGAATACCCATCAA-BHQ1
CBUA0025-F	TCACACTCGACTCTCAGCCATT
CBUA0025-R	TGACTGACGAAGAAGCAGCATT
CBUA0025-probe	FAM-CGATGCGCCAATTTTTTGGTTTTTTACC-BHQ1
CBUA0027-F	GTGCCTCGAGGGTGTTTCTC
CBUA0027-R	CCTCGTGAAATTAAGCGCATTC
CBUA0027-probe	FAM-TTTGCAAAAACGGCCTGGCTCACT-BHQ1
CBUA0034-F	CGTTCAGTAAACGACCTTGTAAACT
CBUA0034-R	CCTCAAATACAAAAATCGTAGCACT
CBUA0034-probe	FAM-TTTATCGACATCCAAATTCCCACAGCC-BHQ1
CBU0334-F	AAAAGGCGGTCACTTAATTGGTTC
CBU0334-R	GCGCTACTGATTGCTGAGCTT
CBU0334-probe	FAM-CATACAAAAATACCCAAAACACGGGTGCG-BHQ1
cbuA0027, cbuA0028 and cbuA0028G C. burnetii expression vector primers	
ArgGH-forminiTn7-F	GGGGTTCGAGGTCGACTTAGCCTCCTTTTAATAATTCATTCAGTG
ArgGH-forminiTn7-R	TATCGATACCGTCGACATGGCTTCGTTTCGCAGCGAAC
LacIO-forminiTn7-F	AATGGAATTCCTCGAGCACTGTCAGGTGCGGG
LacIO-forminiTn7-R	TGCAAGGCCTCCCGGGTTTCCTGTGTGAAACCTGCTG
CBUA0027-forminiTn7-F	ACACAGGAAACCCGGGGTGATGAAAGATAAAAAAAAGAAAAG
CBUA0027-forminiTn7-R	TGCAAGGCCTCCCGGGCTAAGCGATGGCTTGAAG
ProBA-forminiTn7-F	GGGGTTCGAGGTCGACTCAATCTCTAATTTGTCCCG
ProBA-forminiTn7-R	CGCTGCGAAACGAAGCCATGTCGACGGTATCGATA
TetRA-forminiTn7-F	AATGGAATTCCTCGAGTTAAGACCCACTTTCACATTTAAG
TetRA-forminiTn7-R	GGTTGGCCTGCAAGGCCTCCCGGGCTTTTCTCTATCACTGATAGGG
CBUA0028-forminiTn7-F	TAGAGAAAAGCCCGGGATGAGGATATTTAAAACACGCTATTTC
CBUA0028-forminiTn7-R	CTGATAGAGGTGCTGTGACCCGGGAGGCCTTGCA
CBUA0028G-forminiTn7-F	TAGAGAAAAGCCCGGGATTCTGGGATATTCCTTTATCTATAAAAAAC

CBUA0028G-forminiTn7-R	TGCAAGGCCTCCCGGGTCACAGCACCTCTATCAGTTC
cbuA0027, cbuA0028 and cbu0665 cell-free expression vectors	
CBUA0028-forpET-F	AGGAGATATACCATGAGGATATTTAAAACACGCTATTTC
CBUA0028-forpET-R1	GGCTTACCTTCAAGCTTCGAATTATCAACCACTTTGTACAACAGCACCTCTATCAGTTCG
CBUA0028-forpET-R2	GGTGGTGGTGCTCGAGACGCGTAGAATCGAGACCGAGGAGAGGGTTAGGGATAGGCTTACCTTCAAGCTTCG
CBUA0027-forpDEST15-F	CAAACAAGTTTGTACAAAATGAAAGATAAAAAAAAGAAAAGTAAAACTC
CBUA0027-forpDEST15-R	CAAACCACTTTGTACACTAAGCGATGGCTTGAAGG
CBU0665-forpEXP1-F	CAAACAAGTTTGTACAAAATGCTTTTAAGAGGAATAATAGTG
CBU0665-forpEXP1-R	CAGCCGGATCAAGCTTCTATTGAAGTCTGGTTAAACC
EMSA promoter primers	
PCBUA0028-EMSA-F	ATGATCTTGCCAAAGCGGG
PCBUA0028-EMSA-R	TTTTCTGATTTTGCCCATCGATG
PCBUA0029-EMSA-F	GATCTTAACCAACCTCAGAATC
PCBUA0029-EMSA-R	TTTAAGAGTGTGGGCTGG
PGroES-EMSA-F	TTTTGCCACCAGCCGTTAATTC
PGroES-EMSA-R	TCTTCAAGGCGACGGAC
cbuA0028 and cbuA0027 promoter analysis vectors	
LysCA-forminiTn7-F	GGGGTTCGAGGTCGACTTATTCCAGAACTATTTCCTGAG
LysCA-forminiTn7-R	TATCGATACCGTCGACATGGCTTCGTTTCGCAGCG
NoP-mScarlet-i-F	TTACTCAATGGAATTCATGGTGAGCAAGGGCGAG
NoP-mScarlet-i-R	GCCCAAGCTTCTCGAGCTAAACAAAAAACCCTGTCACTTTTTAC
PCBUA0027-Frag1-F	TTACTCAATGGAATTCATGAGGATATTTAAAACACGCTATTTC
PCBUA0027-Frag1-R	TGCTCACCATAAGCTTCCTCTATCAGTTCGCCTATTTTTAATAATG
PCBUA0027-Frag2-F	TTACTCAATGGAATTCTACACGATGGAGATTTAGGTTC
PCBUA0027-Frag2-R	TGCTCACCATAAGCTTCCTCTATCAGTTCGCCTATTTTTAATAATG
PCBUA0027-Frag3-F	TTACTCAATGGAATTCAGAAGATAAAGCTTTTTTTGTATATGGTTATG
PCBUA0027-Frag3-R	TGCTCACCATAAGCTTCCTCTATCAGTTCGCCTATTTTTAATAATG
mScarlet-i-forPCBUA0027-F	AAGCTTATGGTGAGCAAGGGCGAG
mScarlet-i-forPCBUA0027-F	GCCCAAGCTTCTCGAGCTAAACAAAAAACCCTGTCACTTTTTAC
PCBUA0027-Frag4-mScarlet-i-F1	GATATGAAAGAATTAGAGCTTCAATCATTATTAAAAATAGGCGAACTGATAGAGGAAGCTTATGGTGAGCAAGGGCGAG
PCBUA0027-Frag4-mScarlet-i-F2	TTACTCAATGGAATTCGTACAAAAAGTTGTCAAAAACTTATTTTGATATGAAAGAATTAGAGCTTCAATC
PCBUA0027-Frag3-mScarlet-i-R	GCCCAAGCTTCTCGAGCTAAACAAAAAACCCTGTCACTTTTTAC
PCBUA0028-mScarlet-i-F1	GTACAGATTGACAAATAGTTCACTTAGTGATATAGTCTTAACAATAAGGGAAAGCTTATGGTGAGCAAGGGCGAG
PCBUA0028-mScarlet-i-F2	TTACTCAATGGAATTCCTGATGGAAATCTGCTCGAGATAGGCAATGAGTACAGATTGACAAATAGTTCACTTAG
PCBUA0028-mScarlet-i-R	GCCCAAGCTTCTCGAGCTAAACAAAAAACCCTGTCACTTTTTAC

Supplemental file 1 – Construction of plasmids used in this study.

Construction of pB-TyrB-QpH1ori

PCR was carried out to amplify the pBR322 origin of replication, *tyrB* gene from *E. coli*, and *1169^P*-CAT-*sacB* fragment. These fragments were cloned using In-Fusion HD to create pB-TyrB. The QpH1 replication machinery and origin of replication (*cbuA00036*-*cbuA0039a*) was amplified from the QpH1 plasmid and cloned into pB-TyrB digested with SbfI using In-Fusion HD to create pB-TyrB-QpH1ori.

Construction of CRISPRi plasmids

The *proBA* genes were amplified from *Legionella pneumophilia* JR32 genomic DNA (gDNA) and fused to the *cbu1169* promoter by PCR. The *1169^P-proBA* fragment was cloned into AvaI digested pJMP1356 by In-Fusion HD to create pB-CRISPRi. Single guide RNA (sgRNA) targeting sequences were generated by annealing two complementary 24 bp oligonucleotides, resulting in a 20 bp double stranded DNA (dsDNA) fragment with a 4 bp BsaI-compatible overhang on the 5′ ends. The sgRNA target dsDNA sequences were cloned into pB-CRISPRi digested with BsaI using the NEBridge Golden Gate Assembly kit (BsaI-HF®v2) kit.

Construction of CRISPRi complementation vectors

The targeting regions chosen for CRISPRi knockdown of *cbu1624* and *cbuA0027* were codon optimized using overlapping oligonucleotides to add silent mutations to the *cbu1624* (*cbu1624*-CO) and *cbuA0027* (*cbuA0027*-CO) sequences. The *cbu1624*-CO and *cbuA0027*-CO codon optimized genes were amplified by PCR and cloned into pJB-*lysCA-tetRA* digested with PstI/SalI to create pJB-*lysCA-tetRA-cbu1624*-CO and pJB-*tetRA-cbuA0027*-CO, respectively, using In-Fusion HD.

Construction of *cbuA0027* and *cbuA0028 C. burnetii* expression vectors

The *cbuA0027* and *cbuA0028* or *cbuA0028G* genes were amplified by PCR from *C. burnetii* NMII or G Q212 gDNA, respectively. The *cbuA0027* PCR fragment was then cloned into pMiniTn7T-*argGH-lacIO* digested with SmaI using In-Fusion HD to create pMiniTn7T-*argGH-lacIO-cbuA0027*. The *cbuA0028* and *cbuA0028G* PCR products were cloned into pMiniTn7T-proBA-TetRA digested with SmaI to create pMiniTn7T-*proBA-tetRA-cbuA0028* and pMiniTn7T-*proBA-tetRA-cbuA0028*G.

Construction of *cbuA0027*, *cbuA0028* and *cbu0665* cell-free expression vectors

The *cbuA0027* and *cbu0665* genes were amplified by PCR from NMII gDNA and the resulting PCR products were cloned into BsrGI-digested pDEST15 or pEXP1-DEST to create pDEST15-*cbuA0027* and pEXP1-*cbu0665*, respectively. The *cbuA0028* gene was amplified by PCR from NMII gDNA using oligonucleotides that incorporate a C-terminal V5 tag. The resulting *cbuA0028*-V5 PCR fragment was cloned into pET28a(+) digested with NcoI/XhoI using In-Fusion HD to create pET28a(+)-*cbuA0028*.

cbuA0028 and *cbuA0027* promoter analysis vectors

The promoterless *mScarlet-i* gene was amplified by PCR from pmScarlet-i_C1 (Addgene #85044) and the resulting fragment cloned into EcoRI/XhoI-digested pMiniTn7T-*lysCA* using In-Fusion HD to create pMiniTn7T-*lysCA*-NoP-*mScarlet-i*. The *cbuA0027* promoter fragments 1-3 were amplified by PCR from QpH1 plasmid DNA. The *mScarlet-i* gene was amplified from pmScarlet-i_C1 and cloned with the *cbuA0027* promoter fragments 1-3 into EcoRI/XhoI-digested pMiniTn7T-lysCA using In-Fusion HD to create pMiniTn7T-*lysCA*-P*cbuA0027*-Frag-1-*mScarlet-i*, pMiniTn7T-*lysCA*-P*cbuA0027*-Frag-2-*mScarlet-i* and pMiniTn7T-*lysCA*-P*cbuA0027*-Frag-3-*mScarlet-i*. The *mScarlet-i* gene was fused by PCR to the *cbuA0027*-Frag-4 and *cbuA0028* promoters using overlapping oligonucleotides. The resulting *cbuA0027*-Frag-4-mScarlet-i and *cbuA0028*-mScarleti-I PCR products were cloned into EcoRI/XhoI-digested pMiniTn7T-lysCA using In-Fusion HD to create pMiniTn7T-*lysCA*-P*cbuA0027*-Frag-4-*mScarlet-i* and pMiniTn7T-*lysCA*-P*cbuA0028*-*mScarlet-i*, respectively.
